# Fruit thinning techniques incorporated with calcium boron or potassium phosphite for improving Medjool date palm fruit quality under semi-arid regions

**DOI:** 10.1038/s41598-026-55435-7

**Published:** 2026-06-18

**Authors:** Samy El-Kosary, Ahmed A. Rashedy, Sherif El-Sharabasy, Ahmed Abd-Allah, Ashraf E. Hamdy

**Affiliations:** 1 Department of Pomology, Faculty of Agriculture, Cairo University, Giza, Egypt; 2 Central Laboratory for Date Palm Research and Development, Agricultural Research Center, Gamaa St, Giza, 12619 Egypt; 3 Department of Horticulture, Faculty of Agriculture, Al-Azhar University, Cairo, 11884 Egypt

**Keywords:** *Phoenix dactylifera* L., Sugars, Kimri stage, Khalal stage, Bunch, Strand, Fruit set, Biochemistry, Plant sciences

## Abstract

Low fruit quality presents a significant challenge for numerous date-producing countries, including certain cultivars resulting from tissue culture. Fruit thinning alone enhances fruit quality but slightly decreases productivity. So, the aim of this experiment was to improve the quality characteristics of Medjool fruits through four different fruit thinning methods: control (12 bunches per palm), removing 1/3 bunches number, removing 1/3 strands length of the bunch, removing 1/3 strands number of the bunches, these methods were integrated with spraying 0.25% calcium boron (CaB) or 0.2% potassium phosphite (KP), as well as control (tap water), which applied once (fruit set or Kimri stage or Khalal stage) for thinning methods. At Kimri stage, removing 1/3 strands number successfully enhanced fruit weight, length, and size by 10.44%, 8.28%, and 15.68%, while incorporating CaB to removing 1/3 strands number led to increases of 43%, 26.78%, and 52.78%, while incorporating KP with removing 1/3 strands number resulted in increases of 42.67%, 26.31%, and 44.22%, compared to control, as mean of both seasons, respectively. At Khalal stage, combining removing 1/3 strands number and CaB increased fruit TSS and total sugars by 18.85% and 33.65%, while combined removing 1/3 strands number and KP increased them by 19.29% and 23.81%, respectively. In conclusion, nutrient spraying improved fruit chemical properties and the early spraying stage was more effective in improving fruit physical properties, whereas later spraying improved fruit chemical properties. Also, thinning enhances fruit physical properties and provides more nutrients for the remaining fruits. The integrated application of thinning and spraying nutrients improved fruit’s physical and chemical properties.

## Introduction

Egypt ranked the world’s top producer of dates, contributing approximately 1,867,064 tons, from more than 16 million date palm trees, which representing one-fifth (19%) of the global production and 24% of the Middle Eastern production^1^. Date palm is distributed in many parts of Egypt, such as desert areas, oases and the Nile valley^2^. Palm trees can grow well in harsh environmental and climatic conditions which are unsuitable for most types of fruits. Date palm cultivars are classified into three main categories based on fruit moisture content: dry, semi-dry, and soft cultivars^3^. Date palms can thrive in harsh conditions and are the predominant trees in arid and semi-arid regions, contributing significantly to intercropping systems and ecosystem stability^4^. Medjool cv. is regarded a highly valuable variety of dates in European trade due to its quality fruits, international market value and high yields^5,6^, along with high nutritional values and bioactive compounds^7^. However, many date-producing countries face the significant challenge of low fruit quality, even in some cultivars derived from tissue culture. These lower-quality fruits are often sold locally at discounted prices.

Thinning is a common practice widely used in horticultural production that reduces the total number of fruits to enhance the weight, size, and nutritional quality of the remaining fruit by reducing their competition for water and food competition. Madani et al.^8^ reported that, early thinning (during pollination) of Piyarom date palm was more effective than later thinning (Kimri) in increasing fruit weight, while, later thinning (kimri) increased ascorbic acid content and the percentage of Tamar. Removing 1/3 of terminal tips of central strands in Kimri stage of Piyarom date palm significantly improved fruit TSS compared to removing 1/3 of total strands from terminal tips during pollination^8^. Retaining 8–10 fruit bunches per palm recorded the highest productivity and fruit quality compared to 12 bunches per palm in Sukary date palm fertilized with 5 kg sulfate of potash per palm^9^.

Several studies have highlighted the significance of using macro and micronutrients to enhance the yield and quality of date palm fruits. Potassium (K), known for its role in fruit quality attributes, plays a crucial part in regulating the movement of photosynthetic products and activating enzymes^10^. K has a vital role in fruit set, production and fruit quality^11^. Since it regulates a various of biochemical and physiological processes inside plants. Also, K plays a necessary in role amino acid synthesis, sugar movement and assimilate, subsequently leading to accumulation of carbohydrates^12,13^. Moreover, K regulates carbohydrate biosynthesis, cell water content, nitrogen uptake and translocation^13–15^. Studies have shown that applying 1% to 2% K_2_SO_4_ at the Khalal stage for the Shahany date palm variety can lead to an increase in fruit length, diameter, TSS%, and yield^16^. Furthermore, potassium citrate at 0.5%, combined with 0.2% methionine improved fruit quality of Balady mandarin^17^. Phosphite also acts as anti-fungal and antibacterial properties and maintain fruit quality during cold storage^18,19^.

Calcium (Ca) is regarded as a vital nutrient in managing fruit quality^20–23^. It plays a crucial role in cell wall structure maintenance and stabilizing cell membranes^24^. Ca has been shown to prevent fruit abscission, delay fruit senescence and enhance fruit firmness^25,26^. Also, Ca forms calcium pectate in the cell walls after interacting with pectic acid which safeguards cell walls from enzymatic degradation^27^. It improves fruit total soluble solids (TSS) and fruit firmness^23,28^. Furthermore, Ca improves fruit firmness, prolongs fruit shelf-life, increases ascorbic acid content, and reduces storage breakdown, rotting, and browning in apple fruit^21^.

Foliar application of Ca and B was more effective than soil application^29–31^. B increases fruit sugar content by enhancing the transport of carbohydrates through the association of OH groups in sugars with the borate radical, promoting their movement into the plant, which resulted in increased fruit TSS and sugar content as well as increased fruit dry matter^32,33^. Foliar application of 1500 ppm B at the Khalal and Rutab stages increased dry matter and TSS of Halawi and Sayer date fruits^34^. Also, Sobeih^35^ reported that, spraying Sayer date palm trees with 300 ppm B significantly increased fruit weight while reducing fruit water content. While, thinning fruit could reduce yield, so applying nutrients might help alleviate this issue by enhancing fruit quality. Hence, this study was aimed to improve the quality characteristics of Medjool fruits under Bahariya Oasis conditions using integrated management strategies that included different thinning methods (removing number of bunches from date palm tree or removing parts of strands within the bunches) and spraying nutrients (Ca with B and K with phosphite) at three different stages of fruit growth (fruit set stage, Kimri stage and Khalal stage).

## Materials and methods

This experiment was conducted during 2019 and 2020 seasons on seven-year-old Medjool date palm cultivar. Medjool cultivar was produced from tissue culture techniques and grown under Bahariya Oasis latitudes 31° and 31.5°, and longitudes 28.3 and 29.1°) Giza, Egypt. The authors confirm that all experiments were performed in accordance with relevant named guidelines and regulations.

### Preparing date palm trees to apply treatments

The number of bunches was standardized to 12 bunches per palm. The orchard soil was sandy and irrigated using a drip irrigation system. Annually agricultural practices were carried out on all the tested date palms^36^. Pollination was done manually using Ghanami pollens source^37^ in the first week of April in both seasons (Pollination once in the Medjool cultivar). This investigation aimed to study the effect of fruit thinning and spraying calcium boron (CaB) and potassium phosphite (KP), as well as their interaction on fruit quality attributes.

### Thinning treatments

**Fig. 1:**
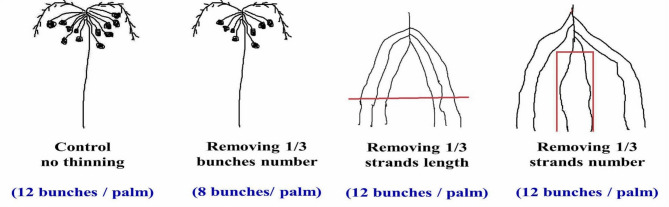
Different thinning methods include control (without thinning =12 bunches per palm), removing 1/3 of the total bunches number (8 bunches per palm), removing 1/3 strands length of the bunches from the base (12 bunches per palm) and removing 1/3 strands number from the center of the bunches (12 bunches per palm).

The fruit thinning treatments (Fig. 1) were carried out after fruit set immediately at the third week of April as follows:

Control date palms were left without thinning which bearing 12 bunches per palm.

Remove 1/3 strand length from the bunches.

Remove 1/3 strand number from the center of the bunches.

Remove 1/3 of the total bunches number (keeping 8 bunches/ date palm).

Removing 1/3 strands number, from the center of the bunches, was carried out by counting the average total strands per bunch, then a third of the total number of the strands was removed from the center of the bunches. Also, removing 1/3 strand’s length from the bunches, was carried out after measuring the average of strand length, then the 1/3 of length was removed from the strand end.

###  Spraying treatments

The integrated spraying substance either 0.25% calcium boron (CaB) (11.4% Ca and 0.7%B from Misr Holland for Investment and Agricultural Development Company, Egypt.) or 0.2% potassium phosphite (KP) (N: P:K 0-20-30 from Agro Gibson Company, Egypt) as well as control (tap water) were applied once with each group of thinning methods on palms after fruit thinning either at the fruit set stage (one month after flowering at third week of April) or at the Kimri stage (four months after flowering at mid of July) or at the Khalal stage (six months of flowering at mid of September). Each treatment was applied on nine date palms, representing three replicates of each treatments in both seasons. Every date palm requires 5 l of solution which is applied once. The sprayed solution just prepared before spraying contains 0.1% (v/v) tween 20 as surfactant and has a pH of 5.8. A row of date palm trees was kept as a border for spraying treatments. The spraying was done in the morning using a small sprayer (20 l).

After fruit set refers to one month after flowering at Hababouk stage. Kimri stage, where the fruit appears green in color and is marked by a rapid increase in volume and weight due to cell division and elongation. Also, in this stage, the fruits are turgid and bitter (rich in tannins) and present high moisture content between 75 and 85% and the end of green color. Khalal stage, which is considered the stage of physiological maturity, the color of the fruit begins to change in color from green to yellow. The increase in fruit weight and volume decreases significantly until the fruit attains the maximum volume.

### Measurements

Medjool date fruits were harvested at Tamr stage (semi-dry) in October. Fruit samples of each replicate for each treatment were randomly taken to assess the fruit characteristics.

#### Bunch weight

Bunch weight (Kg) was weighted after harvesting (kg) for each replicate and the average bunch weight was recorded. Every date palm pruned to contains 12 bunches (36 bunches per replicate) except for treatment of removing 1/3 bunches number which became 8 bunches per date palm tree (24 bunches per replicate).

#### Fruit physical characteristics

Average fruit weight (g): Ten fruits were randomly selected from each bunch in each replicate and weighed to determine the average fruit weight. Seed weight (g): Seeds were separated from the fruits and their average weight was calculated in grams. Fruit diameter and length (mm): A digital caliper was used to measure the diameter and length of ten individual fruits from each replicate. Fruit size (cm³): The volume of fruit samples was determined using water displacement. The fruits were submerged in a graduated cylinder filled with a known volume of water. The difference between the initial water level and the level after submerging the fruits represents the fruit’s volume. The average fruit size was then calculated in cubic centimeters.

#### Fruit chemical characteristics

Total soluble solids % (TSS): A digital refractometer was used to measure TSS in the fruit juice^38^. Moisture content (%): Fruits from each replicate were cleaned, with perianths and seeds removed. The flesh was then cut into pieces and dried at 60–65 °C for 48–72 h until a constant weight was achieved. The moisture content was calculated as the percentage of weight lost during drying compared to the initial fresh weight^38^. Titratable acidity (%): The acidity content, expressed as a percentage of malic acid^38^. Total sugars %: The Smogy method was used to determine the total sugars content (including reducing and non-reducing sugars) as a percentage^38^.

#### Statistical analysis

In each season, the experimental design was factorial, containing 3 factors: thinning method, spraying substance, and spraying stage. The main plot was the thinning method (four levels), the spraying substance was in the sub-plot (three levels), and the spraying stage was in the sub-sub plot (three levels). Each treatment is represented by three replicates with 3 date palms in each one (9 palms in each treatment). The experimental unit includes 3 date palm trees. A row of date palm trees was made as a border to separate the spray treatment between each other. Each date palm tree was thinned to 12 bunches except for the treatment of removing 1/3 bunches number, which contains 8 bunches only. The least significant difference (LSD) test, as described by Snedecor and Cochran^39^, was used to compare the significance of differences between treatments. Each season was statistically analyzed separately.

## Results and discussion

### Bunch weight

**Fig. 2:**
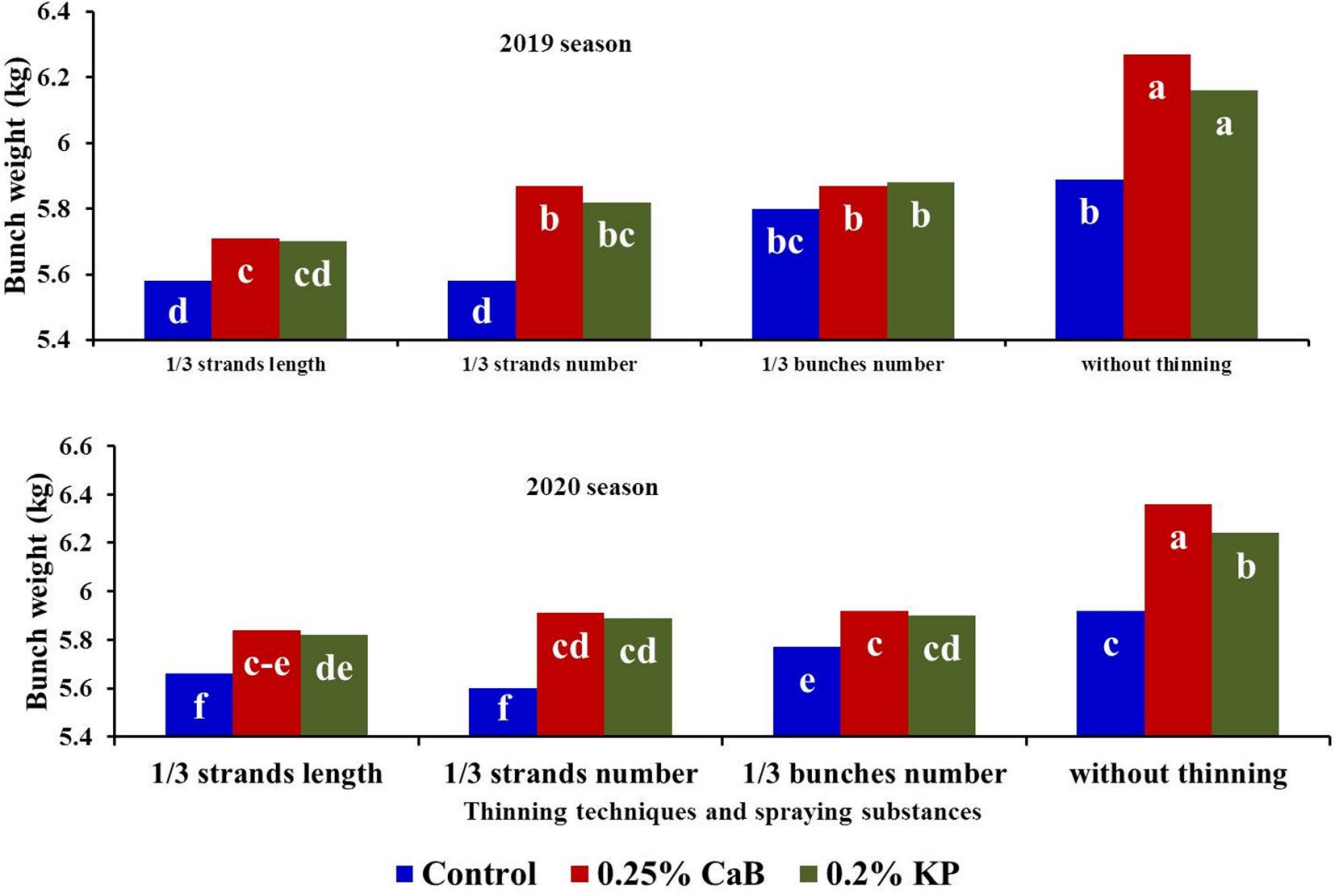
Effect of different thinning techniques (1/3 strands length, 1/3 strands number, 1/3 bunches number and control) combined with spraying substances (0.25% CaB, 0.2 KP and control) on the bunch weight of Medjool date palm during 2019 and 2020 seasons.

For the effect of thinning techniques combined with spraying substances on bunch weight, it can be noticed that control date palms which had no thinning technique (12 bunches per palm) recorded the highest bunch weight (Fig. 2). Removing 1/3 bunches number from semi-dry dates Medjool resulted in a significant increase in bunch weight compared to removing 1/3 strands length. In terms of the effect of nutritional sprays, CaB and KP increased bunch weight compared to the un-sprayed control. The effect of CaB and KP was more pronounced with the removing 1/3 strands number treatment followed by with un- thinned date palms.

**Fig. 3:**
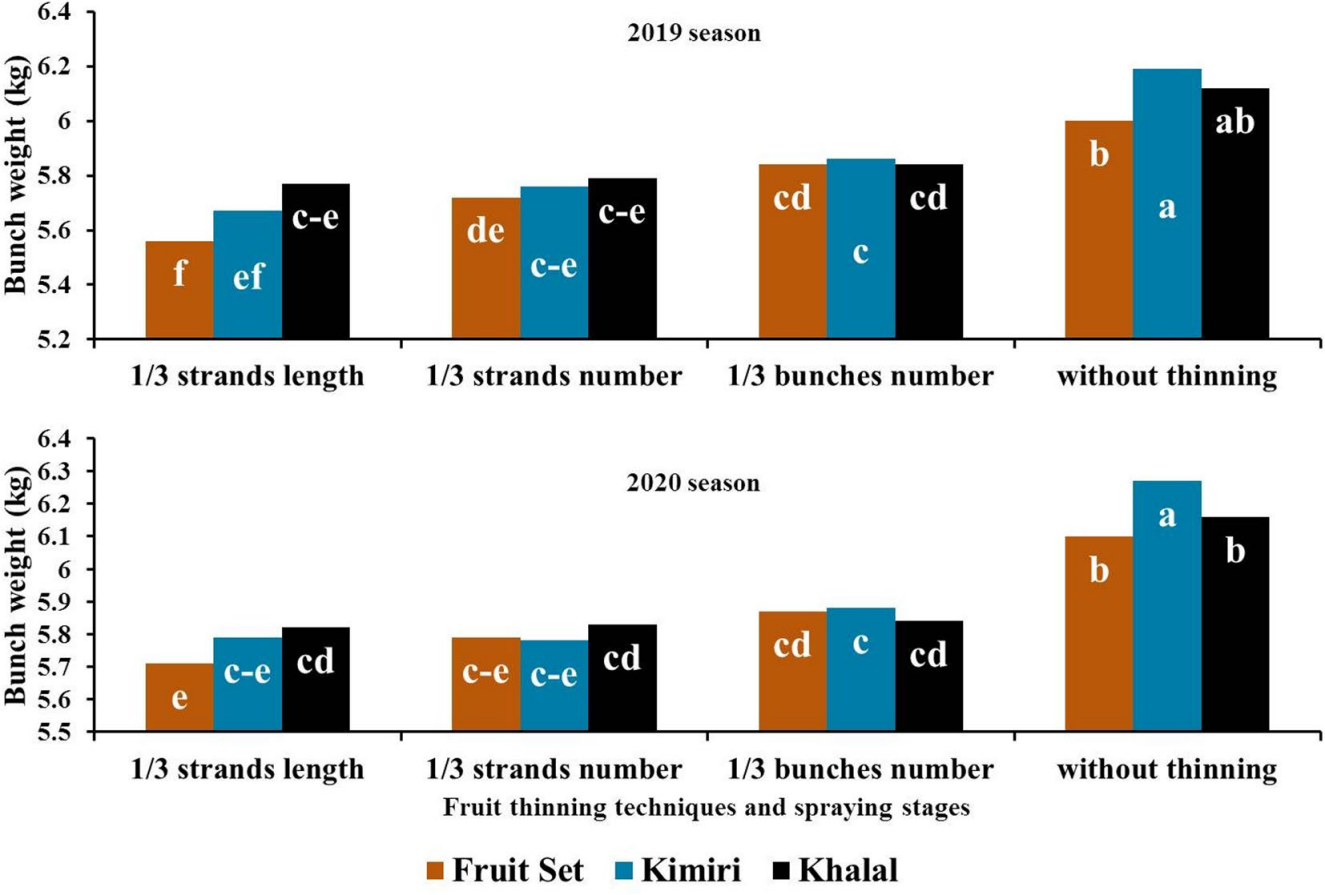
Effect of different thinning techniques (1/3 strand’s length, 1/3 strand’s number, 1/3 bunches numbers and control) combined with spraying stages (fruit set, Kimri and Khalal ) on bunch weight of Medjool date palm during the 2019 and 2020 seasons.

Regarding the effect of the stage on bunch weight (Fig. 3), results indicate that spraying in the later fruit growth stages (Kimri and Khalal stages) significantly increased bunch weight compared to spraying in the early stage (after the fruit set stage).

Concerning the triple interaction (thinning method x spraying substances x spraying stage), the results indicated that non-thinning date palms sprayed with CaB or KP at the Kimri stage exhibited the highest significant bunch weight compared to the other interactions (Table 1). These findings align with Madani et al.^7^, who found that control treatment produced higher bunch weight than the two thinning methods (removing 1/3 of total strands from terminal tips during pollination and removal of 1/3 of terminal tips of central strands) in early Kimri in the Piyarom date palm cultivar. Moreover, Al-hajjaj et al.^40^ found that spraying Medjool fruits with 800 and 1600 ppm potassium increased bunch weight. Also, Al-hajjaj and Ayad^41^ demonstrated that spraying Medjool fruits with 1600 ppm boron increased bunch weight (Table 2).

**Table 1:** Effect of different thinning methods and spraying substances at different fruit growth stages on Medjool bunch weight (kg).

Spraying stages		Spraying substances	Thinning methods by removing					
			1/3 strands length	1/3 strands number		1/3 bunches number	Without thinning	
**Bunch weight (kg) in 2019 season**								
Fruit set		Control	5.43 m	5.47 lm		5.77 f-j	5.80 f-i	
		0.25% CaB	5.67 i-l	5.83 e-i		5.90 e-h	6.17 b-d	
		0.2KP	5.57 j-m	5.87 e-i		5.87 e-i	6.03 c-e	
Kimiri		Control	5.50 k-m	5.47 lm		5.83 e-i	5.90 e-h	
		0.25% CaB	5.70 h-k	5.93 e-g		5.83 e-i	6.40 a	
		0.2%KP	5.80 f-i	5.87 e-i		5.90 e-h	6.27 ab	
Khalal		Control	5.80 f-i	5.80 f-i		5.80 f-i	5.97 d-f	
		0.25% CaB	5.77 f-j	5.83 e-i		5.87 e-i	6.23 ab	
		0.2%KP	5.73 g-j	5.73c-e		5.87 e-i	6.17 b-d	
**Bunch weight (kg) in 2020 season**								
Fruit set		Control	5.57 jk	5.57 jk		5.70 h-j	5.90 e-g	
		0.25% CaB	5.83 e-h	5.90 e-g		5.97 de	6.27 bc	
		0.2%KP	5.73 g-j	5.90 e-g		5.93 ef	6.13 cd	
Kimiri		Control	5.63 i-k	5.47 k		5.80 ei	5.90 e-g	
		0.25% CaB	5.87 e-h	5.93 ef		5.90 e-g	6.53 a	
		0.2%KP	5.87 e-h	5.93 ef		5.93 ef	6.37 ab	
Khalal		Control	5.77 f-i	5.77 f-i		5.80 ei	5.97 de	
		0.25% CaB	5.83 e-h	5.90 e-g		5.90 e-g	6.27 bc	
		0.2%KP	5.87 e-h	5.83 e-h		5.83 e-h	6.23 bc	

The values following the same letter in each season do not differ significantly at a probability level of 0.05. CaB = calcium boron, KP = potassium phosphite.

**Table 2:** Effect of different thinning methods and spraying substances at different fruit growth stages on Medjool fruit weight (g).

Spraying stages	Spraying substances	Thinning methods by removing			
		1/3 strands length	1/3 strands number	1/3 bunches number	Without thinning
**Fruit weight (g) in 2019 season 1st season**					
Fruit set	Control	13.43 g-i	13.90gh	11.73j	12.67 ij
	0.25% CaB	16.03 cd	15.93 cd	15.63de	13.20 hi
	0.2%KP	15.23d-f	17.07bc	16.10 cd	15.33d-f
Kimiri	Control	14.40f	13.63 g-i	13.50 g-i	12.67 ij
	0.25% CaB	15.90 cd	17.50ab	17.50ab	13.83 g-i
	0.2%KP	15.47d-f	17.63ab	15.43d-f	14.40f-h
Khalal	Control	14.40f-h	13.80 g-i	11.73j	13.20 hi
	0.25% CaB	15.73de	18.30a	14.57eg	13.87 g-i
	0.2%KP	15.93 cd	17.70b	15.23d-f	14.00 gh
**Fruit weight (g) in 2020 season**					
Fruit set	Control	13.00qr	14.03 m-q	13.20o-r	13.07p-r
	0.25% CaB	15.30 h-l	17.03b-e	16.83c-f	14.30j-p
	0.2%KP	15.90e-h	16.83c-e	15.90e-h	14.8 h-m
Kimiri	Control	14.33j-o	14.13 l-o	13.50n-r	12.47 r
	0.25% CaB	17.35a-d	18.45a	16.63d-f	14.53i-n
	0.2%KP	16.9 c-f	18.23ab	15.77f-i	14.47j-n
Khalal	Control	14.00 m-q	14.27j-p	14.93 h-m	13.43n-r
	0.25% CaB	17.67a-d	18.00a-c	15.40 g-k	14.17k-q
	0.2% KP	16.63d-f	17.27a-d	15.47 g-j	14.7 h-m

The values following the same letter in each season do not differ significantly at a probability level of 0.05. CaB = calcium boron, KP = potassium phosphite.

Fruit thinning treatments involve removing about 30% of the number of fruits, leading to a reduced yield while improving the quality of the remaining fruit. However, a significant reduction in fruit yield due to thinning will lead to a lower yield. Therefore, thinning technique is important for export, where the price depends on the quality of the fruits. Conversely, the absence of fruit thinning leads to a high yield accompanied with low fruit quality, resulting in a lower price. To achieve a healthy date palm without alternate bearing, sufficient fruit thinning is recommended for optimal results. Since fruit thinning could reduce yield, applying nutrients might help in alleviating this issue.

### Fruit weight (g)

**Fig. 4:**
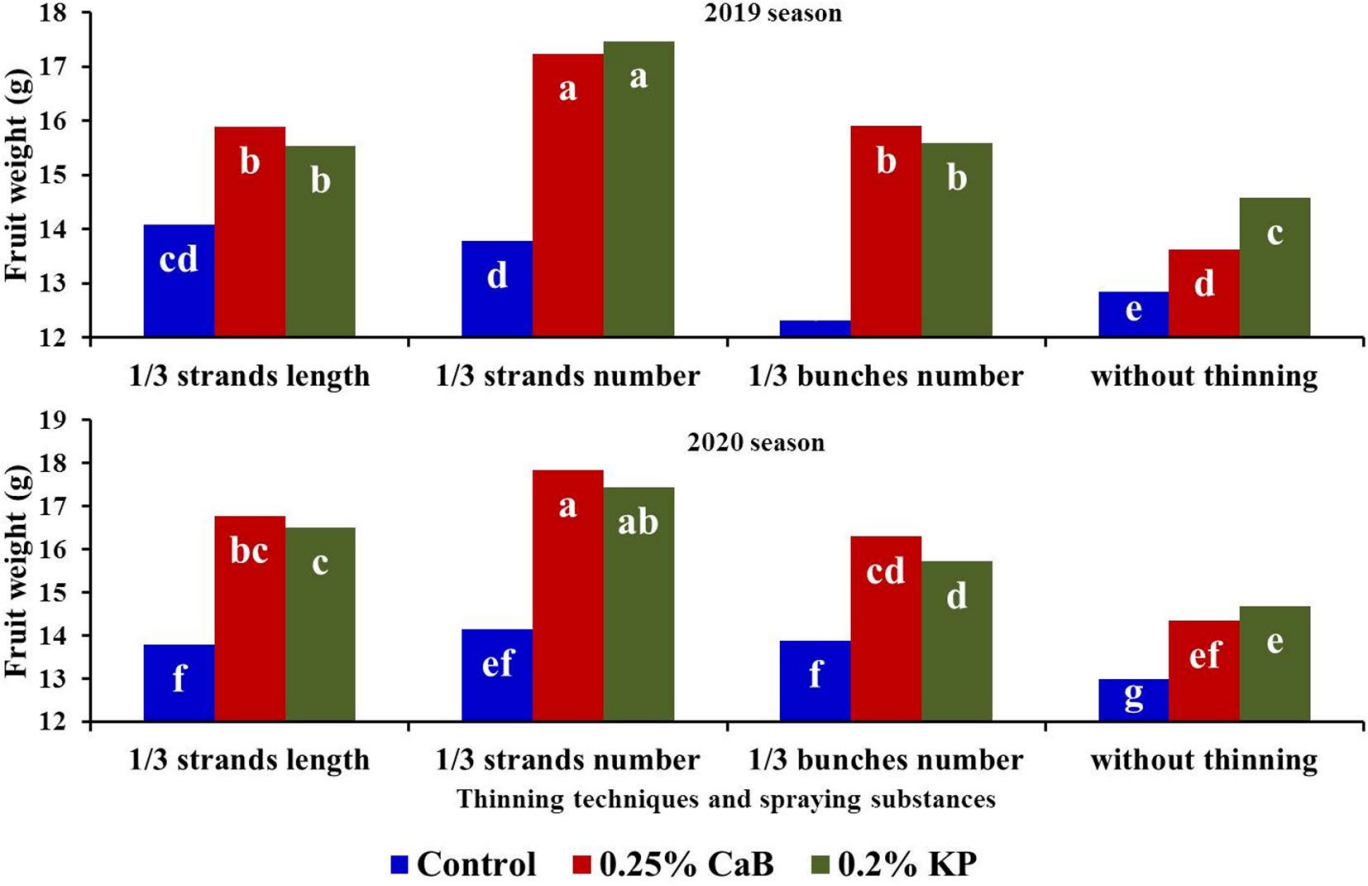
Effect of different thinning techniques (1/3 strands length, 1/3 strands number, 1/3 bunches number and control) combined with spraying substances (0.25% CaB, 0.2% KP and control) on fruit weight of Medjool date palm during 2019 and 2020 seasons.

For the impact of thinning techniques combined with spraying substances on fruit weight (Fig. 4), it can be observed that removing 1/3 strands number followed by removing 1/3 strand’s length as another thinning method, led to a significant increase in Medjool fruit weight compared to other thinning treatments. While, control date palms (without thinning) recorded the lowest fruit weight during the both studied seasons. Concerning the effect of spraying substances, spraying with either CaB or KP resulted in a significant increase in fruit weight compared to the control (Fig. 4) with a pronounced effect when combined with removing 1/3 strands number.

**Fig. 5:**
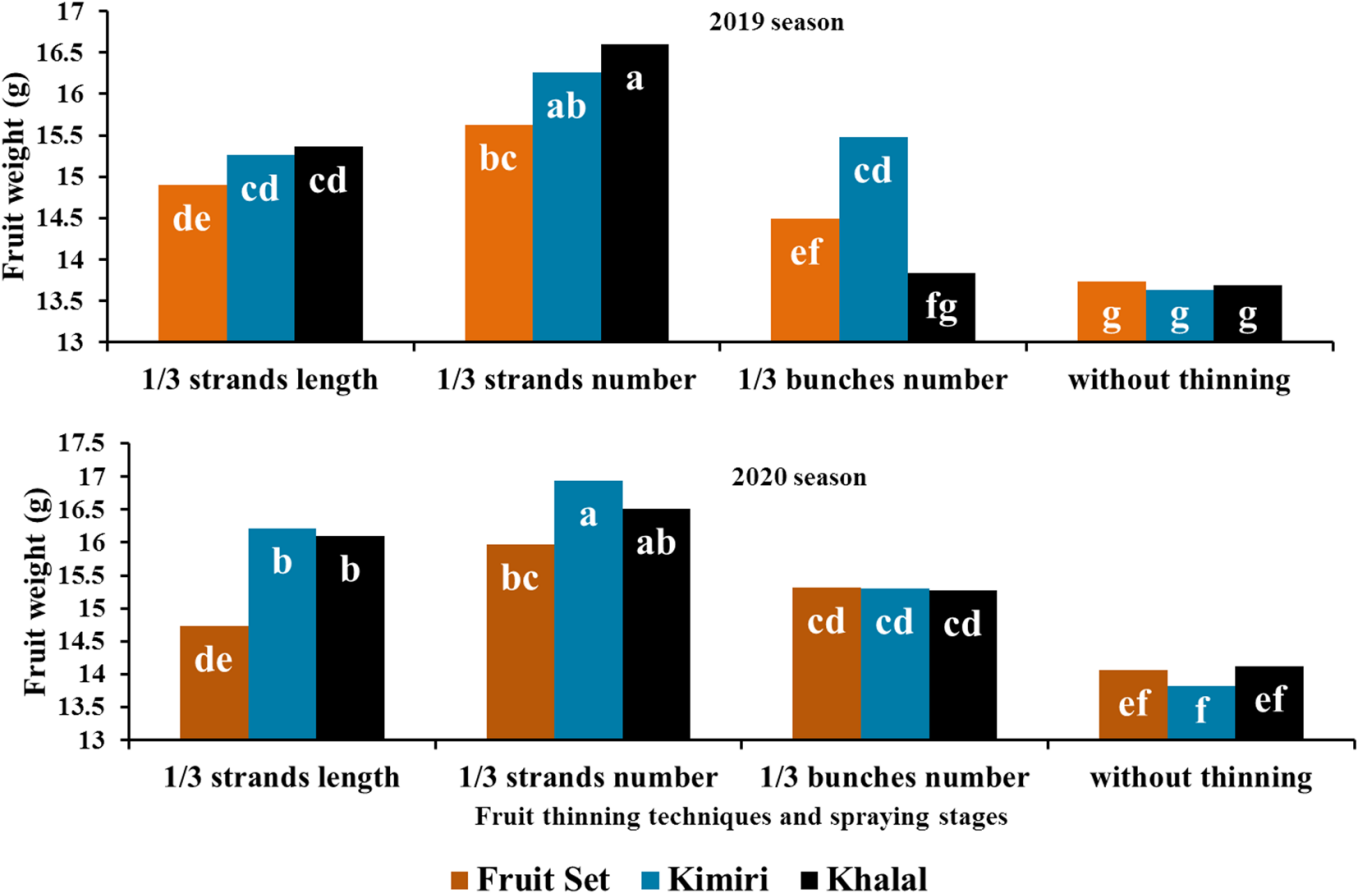
Effect of different thinning techniques (1/3 strands length, 1/3 strands number, 1/3 bunches number and control) combined with spraying stages (fruit set, Kimri and Khalal ) on the fruit weight of Medjool date palm during the 2019 and 2020 seasons.

Regarding the effect of the spraying stage, the data confirm that spraying at the Kimri stage significantly increased fruit weight compared to spraying at the fruit set stage with all thinning treatments (Fig. 5).

Concerning the triple interaction, the data showed that removing 1/3 strands number combined with CaB or KP spraying at the Kimri stage, or spraying with CaB at the Khalal stage, significantly increased fruit weight.

These results were aligned with the findings of El-Assar and Refaat^42^, who found that thinning 40% of bunches led to a significant increase in Sewy fruit weight by 53 and 58% for the first and second seasons, respectively. Also, Al-Hajjaj and Ayad^41^ showed that spraying Medjool fruits with B at 1600ppm increased fruit weight. Al-hajjaj et al.^40^ found that spraying with K increased Medjool fruit weight especially at 800 and 1600 ppm. Foliar application of 1500ppm B at the Khalal and rutab stages increased dry matter and TSS of Halawi and Sayer date fruits^34^. Also, Sobeih^35^ reported that, spraying Al-Sayer date palm trees with 300ppm B significantly increased fruit weight. Furthermore, spraying date palms 1% CaNO_3_ at or 0.5% CaCl_2_ significantly improved fruit weight of Medjool dates^34^. K regulates cytokinin levels which manage the cell division process^43,44^. More recently, Alebidi et al.^45^ reported that a mixture of 2% KNO_3_+1% algae extract led to an increase in the fruit weight of Barhee date fruits. El-kosary et al.^2^ recorded a significant increase in fruit weight of Barhee date fruits due to spraying CaB and Kp incorporated with 30% fruit thinning.

### Seed weight (g)

**Fig. 6:**
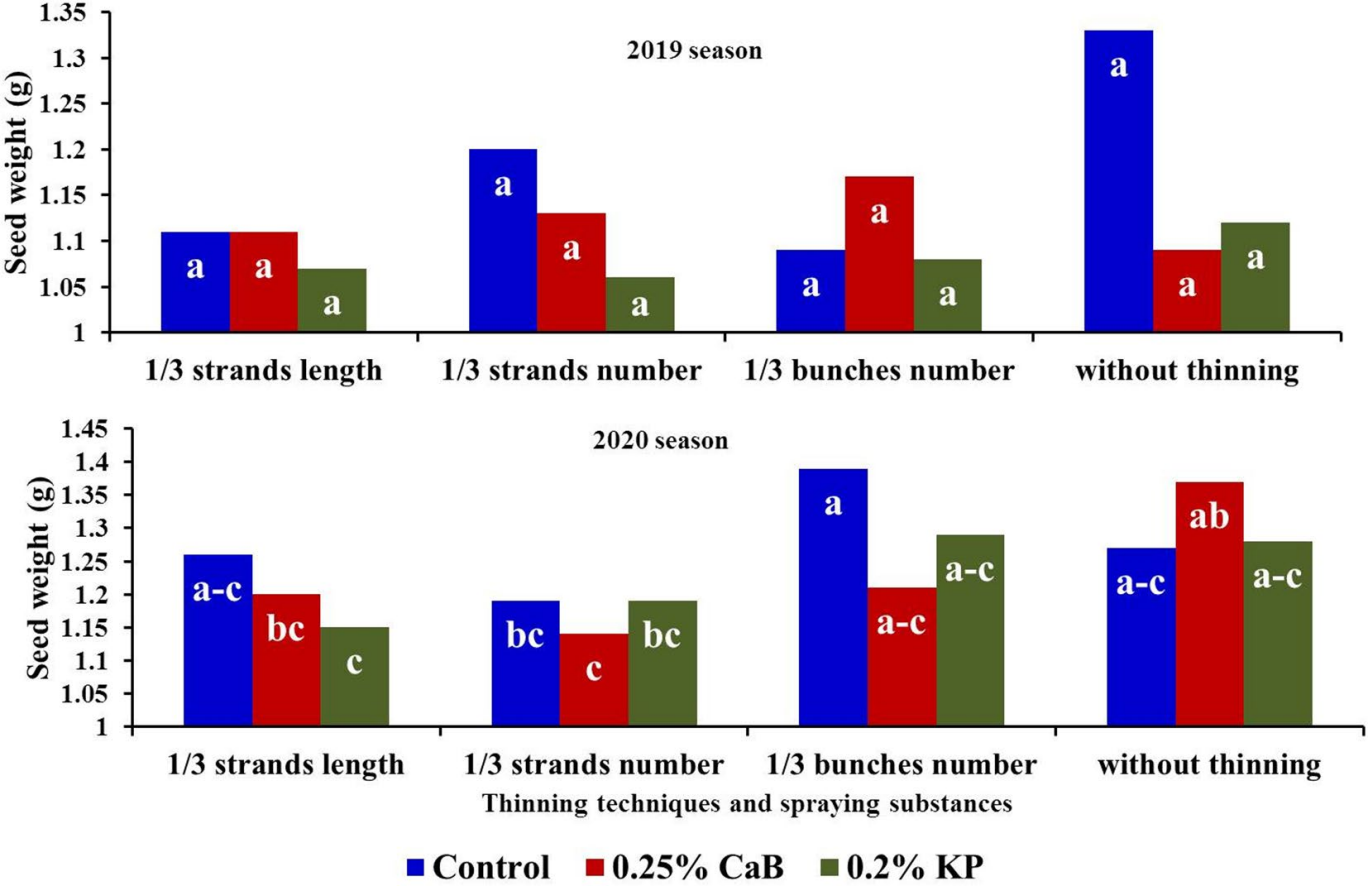
Effect of different thinning techniques (1/3 strands length, 1/3 strands number, 1/3 bunches number and control) combined with spraying substances (0.25% CaB, 0.2% KP and control) on seed weight of Medjool date palm during 2019 and 2020 seasons.

The results indicated that thinning techniques led to a minor reduction in seed weight, especially with removing 1/3 strands length, which became significant in the second season (Fig. 6). Generally, the lowest seed weight values were recorded under spraying Cab and KP substances combined with removing 1/3 strands length or 1/3 strands number (Fig. 6).

**Fig. 7:**
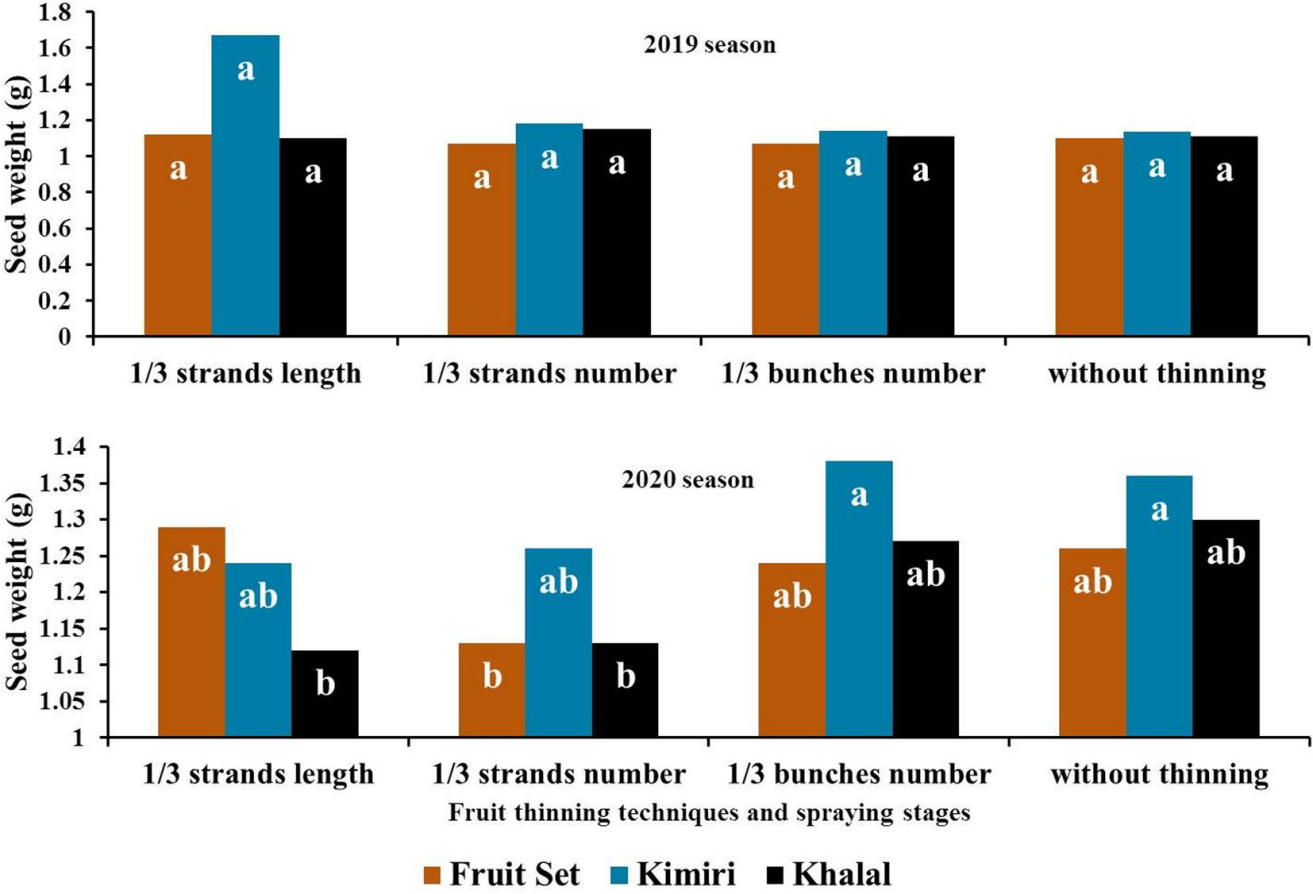
Effect of different thinning techniques (1/3 strands length, 1/3 strands number, 1/3 bunches number and control) combined with spraying stages (fruit set, Kimri and Khalal ) on seed weight of Medjool date palm during 2019 and 2020 seasons.

For the effect of the spraying stage, there is no difference was observed between them on seed weight during both seasons (Fig. 7). Spraying substances did not have a significant effect on seed weight, whereas spraying at Kimri stage tended to increase seed weight slightly without a significant effect.

Regarding the triple interaction, removing1/3 strands number combined with KP spraying after fruit set stages decreased seed weight (Table 3). These results were opposite with Al-hajjaj et al.^40^ who explained that the lowest seed weight in Medjool fruit was recorded by the control and the highest seed weight was observed in foliar potassium application at 800 ppm. Also, Alebidi et al.^45^ recorded an increase in seed weight of Barhee date palms sprayed twice with a mixture of 2% KNO_3_+1%algae extract.

**Table 3:** Effect of different thinning methods and spraying substances at different fruit growth stages on Medjool seed weight (g).

Spraying stages	Spraying substances	Thinning methods by removing			
		1/3 strands length	1/3 strands number	1/3 bunches number	Without thinning
**Seed weight (g) in 2019 season**					
Fruit set	Control	1.20 ab	1.17 ab	1.33 ab	1.13 ab
	0.25% CaB	1.10 ab	1.07 ab	1.33 ab	1.67 ab
	0.2%KP	1.07 ab	0.97 b	0.96 b	1.10 ab
Kimiri	Control	1.07 ab	1.27 a	1.13 ab	1.13 ab
	0.25% CaB	1.00 ab	1.17 ab	1.10 ab	1.10 ab
	0.2%KP	1.13 ab	1.10 ab	1.20 ab	1.17 ab
Khalal	Control	1.07 ab	1.17 ab	1.10 ab	1.13 ab
	0.25% CaB	1.23 ab	1.17 ab	1.17 ab	1.10 ab
	0.2%KP	1.00 ab	1.10 ab	1.06 ab	1.10 ab
**Seed weight (g) in 2020 season**					
Fruit set	Control	1.23 a-e	1.10 b-e	1.33 a-d	1.17 a-e
	0.25% CaB	1.33 a-d	1.23 a-e	1.17 a-e	1.33 a-d
	0.2%KP	1.15 a-e	1.07 c-e	1.33 a-c	1.27 a-e
Kimiri	Control	1.23 a-e	1.17 a-e	1.43 a	1.30 a-d
	0.25% CaB	1.23 a-e	1.23 a-e	1.27 a-e	1.40 ab
	0.2%KP	1.27 a-e	1.37 a-c	1.23 a-e	1.37 a-c
Khalal	Control	1.30 a-d	1.30 a-d	1.40 ab	1.33 a-d
	0.25% CaB	1.03 de	0.97 e	1.20 a-e	1.37 a-c
	0.2%KP	1.03 de	1.13 a-e	1.20 a-e	1.20 a-e

The values following the same letter in each season do not differ significantly at a probability level of 0.05. CaB = calcium boron, KP = potassium phosphite.

### Fruit diameter (mm)

**Fig. 8:**
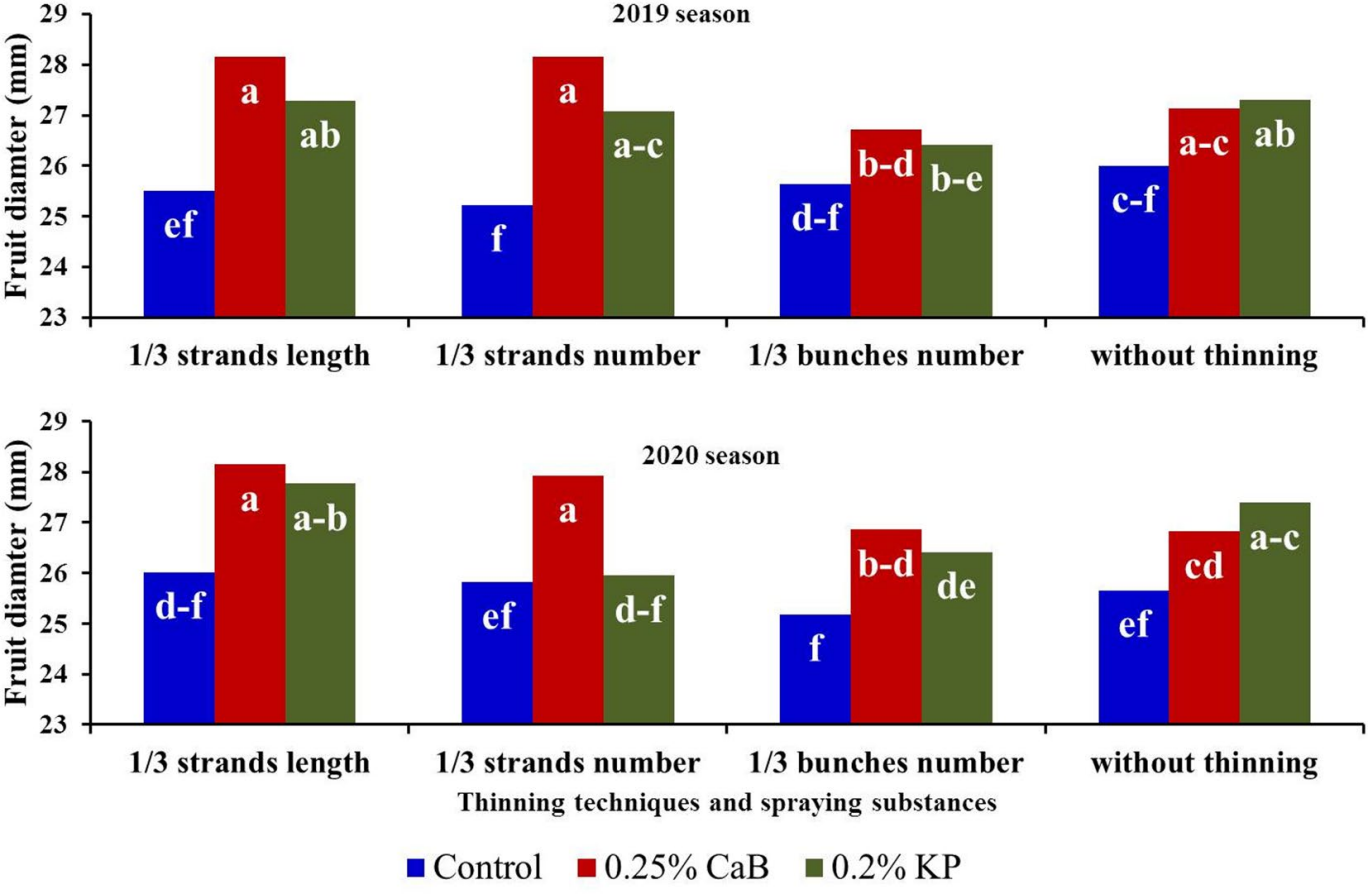
Effect of different thinning techniques (1/3 strands length, 1/3 strands number, 1/3 bunches number and control) combined with spraying substances (0.25% CaB, 0.2% KP and control) on fruit diameter of Medjool date palm during 2019 and 2020 seasons.

For the effect of the thinning technique and the spraying substance, it can be observed that removing 1/3 strand’s length significantly enhanced Medjool fruit diameter compared to removing 1/3 bunches number (Fig. 8). Concerning the effect of spraying substances, spraying CaB or KP significantly increased fruit diameter compared to the control (un-sprayed) in all thinning treatments with more differences when combined with removing 1/3 strands length or 1/3 strands number (Fig. 8).

**Fig. 9:**
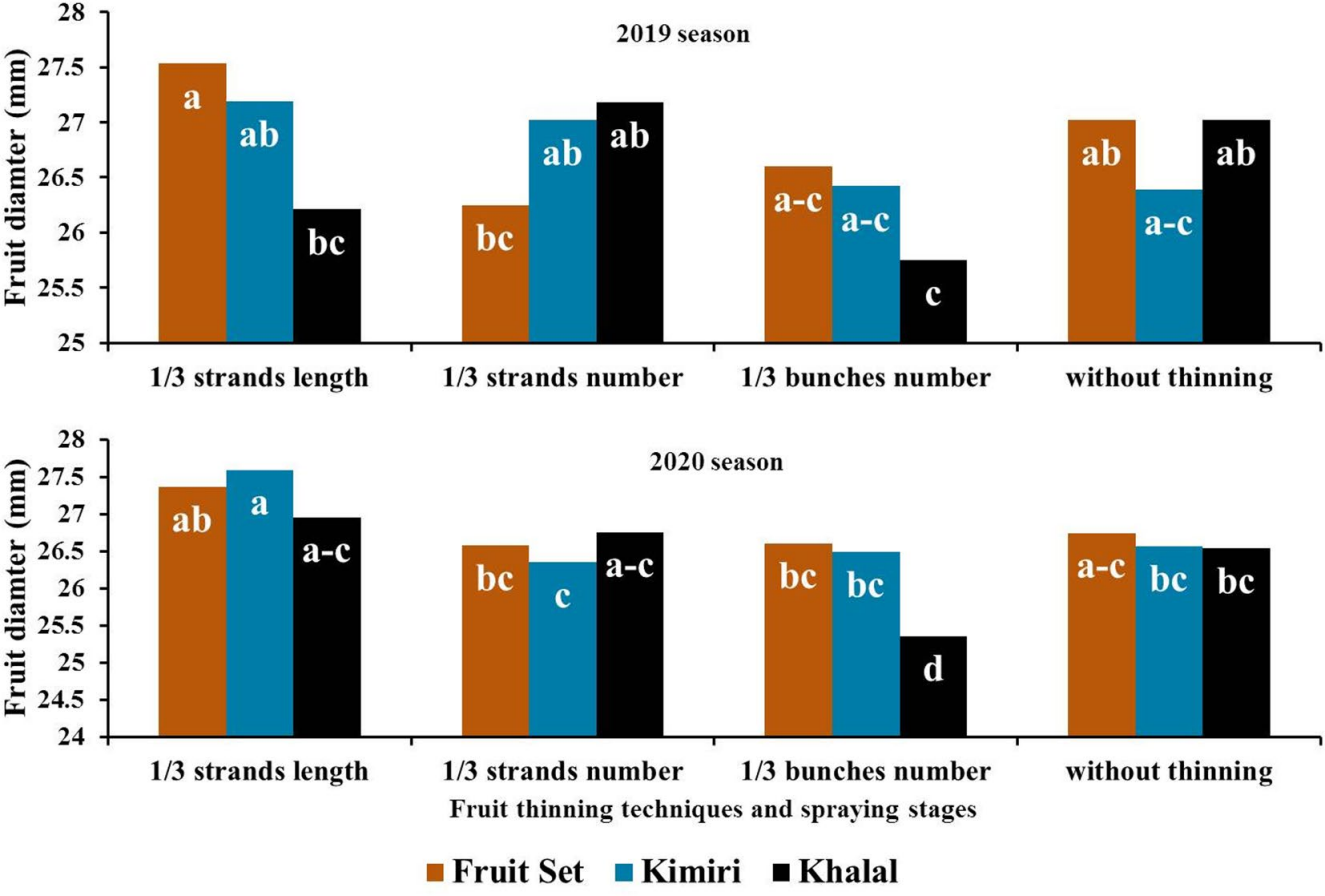
Effect of different thinning techniques (1/3 strands length, 1/3 strands number, 1/3 bunches number and control) combined with spraying stages (fruit set, Kimri and Khalal ) on the fruit diameter of Medjool date palm during the 2019 and 2020 seasons.

As for the effect of the spraying stage, spraying after the fruit set resulted in a slight increase in fruit diameter with no significant differences compared to spraying in Kimri and Khalal stages (Fig. 9). Fruit diameter was increased more when spraying after fruit set, combined with removing 1/3 strands length or 1/3 bunches number (Fig. 9).

With regard to the triple interaction, the data showed that removing 1/3 strands length combined with CaB spraying at the fruit set stage or with KP spraying at Kimri stage increased fruit diameter (Table 4). These results are in agreement with Soliman and Harhash^46^, who found that thinning by removing 15 and 30% of the total number of strands from the center of each bunch led to an increase in fruit diameter of Succary date palm.

**Table 4:** Effect of different thinning methods and spraying substances at different fruit growth stages on Medjool fruit diameter (mm).

Spraying stages	Spraying substances		Thinning methods by removing			
			1/3 strands length	1/3 strands number	1/3 bunches number	Without thinning
**Fruit diameter (mm) in 2019 season**						
Fruit set	Control		25.63 g-j	24.71 j	25.18 h-j	26.1d-j
	0.25% CaB		29.08 a	27.58 a-g	27.16 a-h	27.49a-g
	0.2%KP		27.87 a-f	26.47 c-j	27.46 a-g	27.41a-g
Kimiri	Control		25.24 h-j	26.24 d-j	25.18 h-j	25.90 f-j
	0.25% CaB		27.97 a-e	28.11 a-d	27.88 a-f	26.28d-j
	0.2%KP		28.35 a-c	26.71 c-j	26.21 d-j	26.98b-i
Khalal	Control		25.63 g-j	24.71 j	26.56 c-j	25.93f-j
	0.25% CaB		27.39 a-g	28.78 ab	25.10 ij	27.60a-g
	0.2%KP		25.62 g-j	28.05 a-d	25.60 g-j	27.5 a-g
**Fruit diameter (mm) in 2020 season**						
Fruit set	Control		25.60 g-l	25.25 j-l	25.18 kl	25.77 g-l
	0.25% CaB		28.15 ab	28.01 a-c	27.19 a-g	26.88 a-j
	0.2%KP		28.36 a	26.48 c-k	27.46 a-f	27.61a-d
Kimiri	Control		25.94 e-l	25.25 j-l	25.51 h-l	25.77 g-l
	0.25% CaB		28.50 a	28.27 a	27.75 a-d	26.52c-k
	0.2%KP		28.37 a	25.53 h-l	26.21 d-l	27.43 a-f
Khalal	Control		26.52 b-k	26.96 a-i	24.84 l	25.43 i-l
	0.25% CaB		27.80 a-d	27.50 a-e	25.64 g-l	27.06 a-i
	0.2%KP		26.58 d-k	25.83 f-l	25.60 g-l	27.14a-h

The values following the same letter in each season do not differ significantly at a probability level of 0.05. CaB = calcium boron, KP =  potassium phosphite.

Thabet et al.^34^ reported that foliar spray of 1% CaNO_3_ or 0.5% CaCl_2_ significantly increased fruit diameter of Medjool dates. Also, Alebidi et al.^45^ recorded an increase in Barhee fruit diameter after pollination and one month later with 2% KNO_3_+1%algae extract.

### Fruit length (mm)

**Fig. 10:**
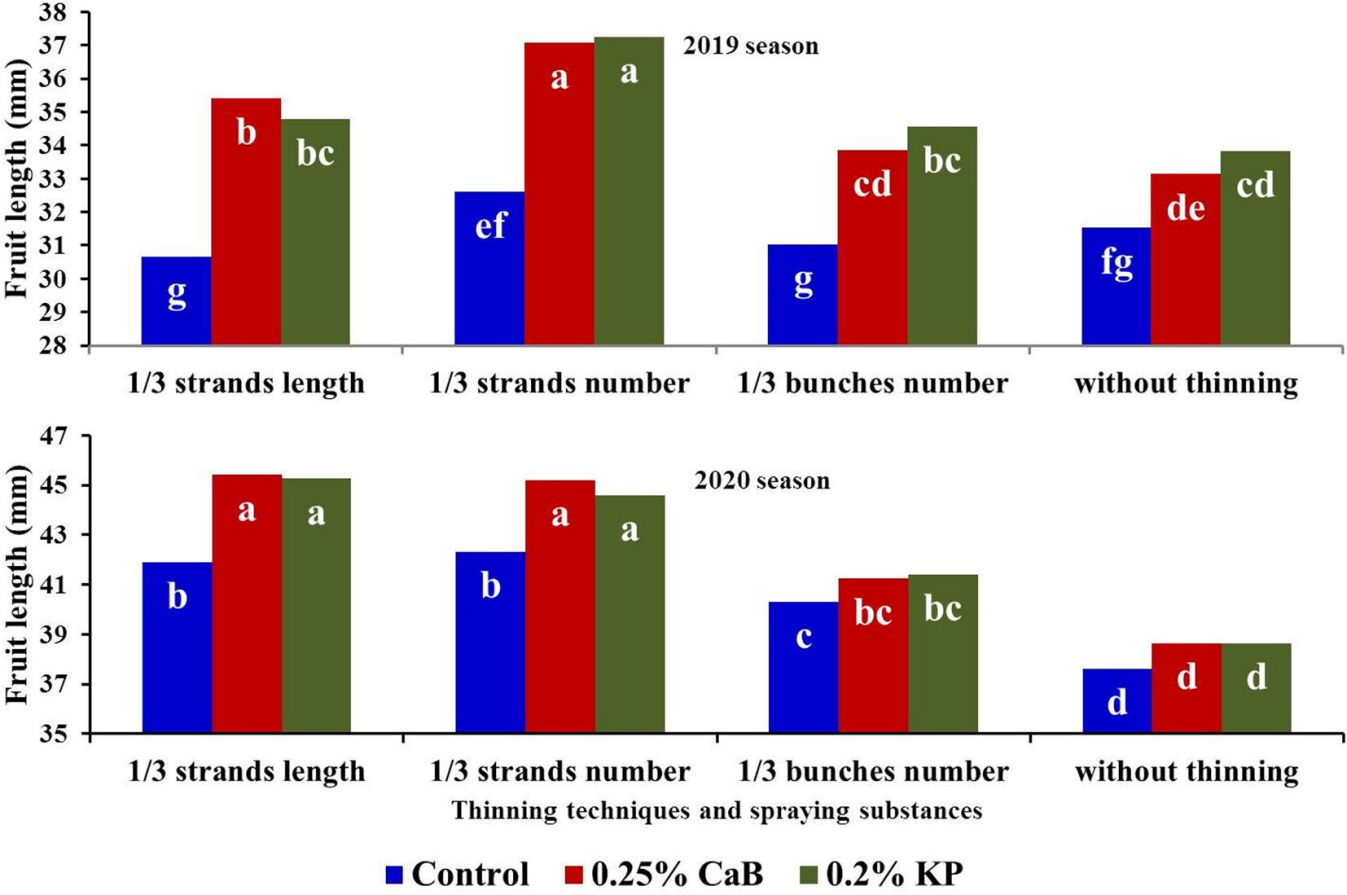
Effect of different thinning techniques (1/3 strands length, 1/3 strands number, 1/3 bunches number and control) combined with spraying substances (0.25% CaB, 0.2% KP and control) on fruit length of Medjool date palm during 2019 and 2020 seasons.

Removing 1/3 strands length and 1/3 strands number thinning methods significantly increased fruit length compared to control treatments (Fig. 10). Regarding the effect of substance, spraying with either CaB or KP significantly increased fruit length compared to the control, and the effect was more pronounced when spraying combined with removing either 1/3 strands length or 1/3 strands number (Fig. 10).

**Fig. 11:**
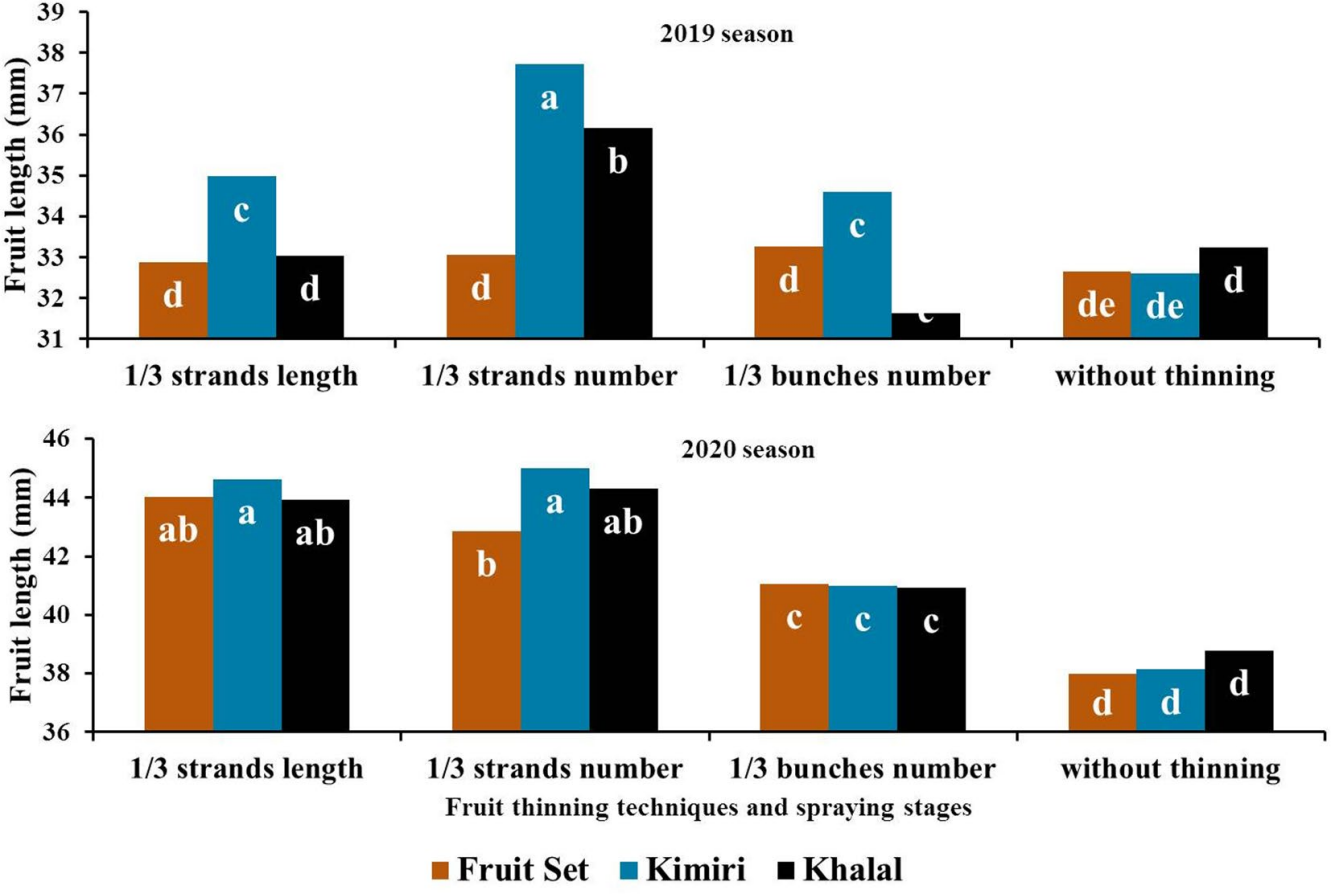
Effect of different thinning techniques (1/3 strands length, 1/3 strands number, 1/3 bunches number and control) combined with spraying stages (fruit set, Kimri and Khalal ) on the fruit length of Medjool date palm during the 2019 and 2020 seasons.

Regarding the effect of spraying stage, spraying at the Kimri stage significantly increased fruit length compared to spraying at the Khalal or after fruit set stages in thinned date palm trees (Fig. 11), while un-thinned control showed no response to spraying treatments, which may be resulting from heavy fruit load which competes for nutrients, water and growth stimulants.

With regard to the triple interaction, the data indicated that removing 1/3 strands number combined with CaB and KP spraying at the Kimri stage increased fruit length (Table 5). These results were consistent with the findings of Soliman et al.^47^ who found that bunch thinning increased the length of Khalas date fruits.

**Table 5:** Effect of different thinning methods and spraying substances at different fruit growth stages on Medjool fruit length (mm).

Spraying stages	Spraying substances	Thinning methods by removing					
		1/3 strands length	1/3 strands number	1/3 bunches number		Without thinning	
**Fruit length (mm) in 2019 season**							
Fruit set	Control	30.14 o	32.09 k-o	31.16 m-o		31.56 l-o	
	0.25% CaB	35.27 d-h	32.87 i-m	33.74 g-k		33.21 i-l	
	0.2%KP	33.24 i-l	34.22 f-j	34.86 e-i		33.19 i-l	
Kimiri	Control	31.52 l-o	32.90 i-m	31.13 m-o		31.14 m-o	
	0.25% CaB	35.70 c-g	39.87 a	35.80 c-f		31.96 k-o	
	0.2%KP	37.68 bc	40.39 a	36.83 b-e		34.71 f-g	
Khalal	Control	30.34 o	32.79 j-n	30.80 no		31.88 k-o	
	0.25% CaB	35.30 d-h	38.51 ab	32.04 k-o		34.25 f-j	
	0.2%KP	33.46 h-l	37.16 b-d	32.03		33.60 h-k	
**Fruit length (mm) in 2020 season**							
Fruit set	Control	41.41 g-m	41.60 f-m	40.03 k-p		37.65 p	
	0.25% CaB	45.07 a-e	42.71 e-k	42.46 e-l		38.23 op	
	0.2%KP	45.59 a-c	44.25 b-f	40.68 h-o		38.10 op	
Kimiri	Control	43.21 c-i	41.67 f-m	40.32 j-p		37.57 p	
	0.25% CaB	45.50 a-d	47.16 a	39.82 l-p		38.24 op	
	0.2%KP	45.17 a-e	46.18 ab	42.80 d-j		38.60 n-p	
Khalal	Control	41.09 g-n	43.71 b-g	40.56 i-o		37.65 p	
	0.25% CaB	45.67 a-c	45.76 a-c	41.45 g-m		39.47 m-p	
	0.2%KP	45.00 a-e	43.42 b-h	40.74 h-o		39.23 m-p	

The values following the same letter in each season do not differ significantly at a probability level of 0.05. CaB = calcium boron, KP = potassium phosphite.

In this regard, spraying Medjool date palms with 1% CaNO_3_ or 0.5% CaCl_2_ significantly increased fruits length^34^. K regulates carbohydrate biosynthesis, cell water content, nitrogen uptake and translocation^13–15^. Studies have indicated that application of 1% to 2% K_2_SO_4_ during the Khalal stage for the Shahany date palm variety increased fruit length, diameter, TSS%, and yield^16^. Thabet et al.^34^ reported that foliar spray of 1% CaNO_3_ or 0.5% CaCl_2_ resulted in a significant increase in the length of Medjool dates.

### Fruit size (cm^3^)

**Fig. 12:**
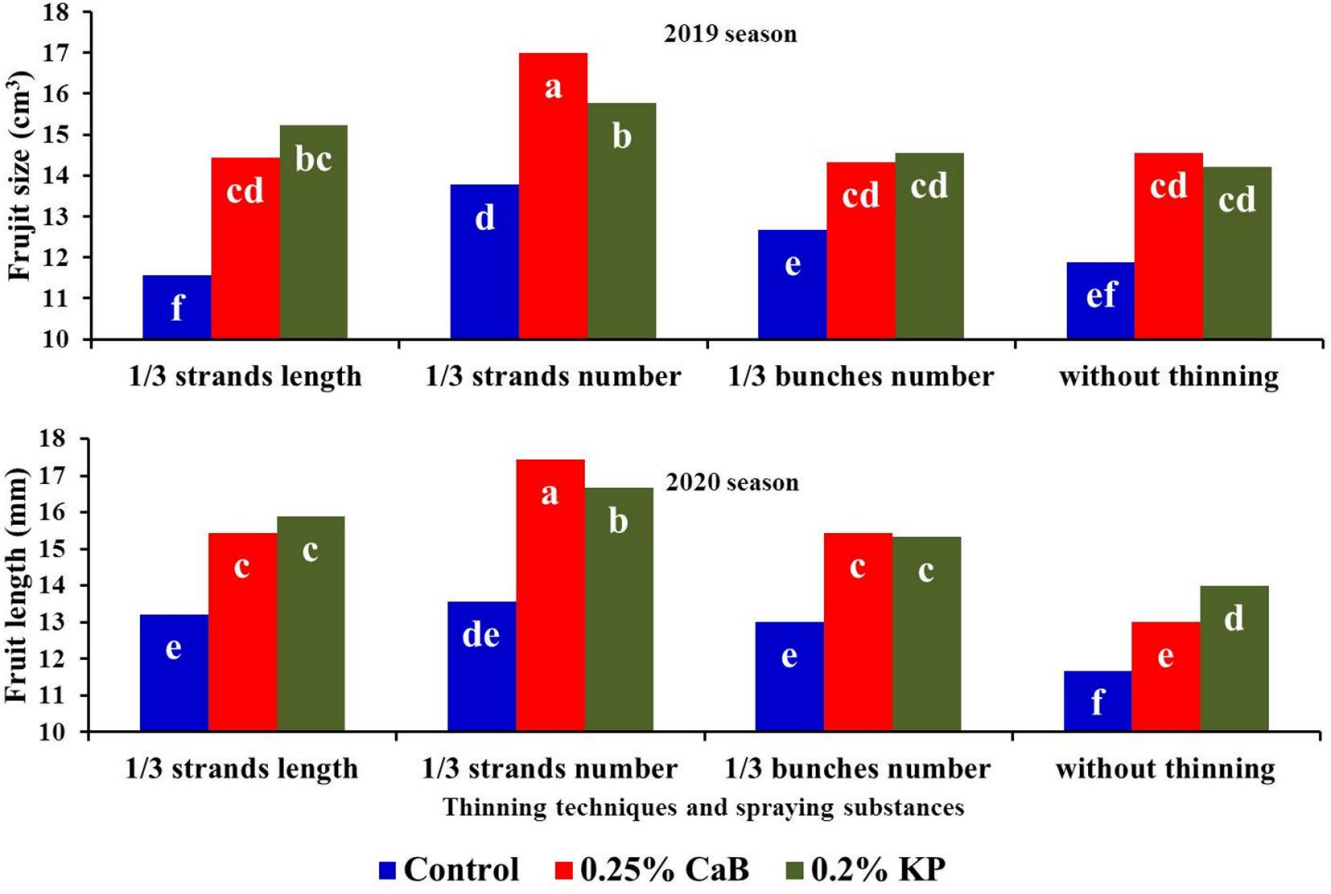
Effect of different thinning techniques (1/3 strands length, 1/3 strands number, 1/3 bunches number and control) combined with spraying substances (0.25% CaB, 0.2% KP and control) on fruit size of Medjool date palm during 2019 and 2020 seasons.

Removing 1/3 strands number followed by removing 1/3 strands length increased fruit size compared to other thinning treatments (Fig. 12). Foliar application of either CaB or KP resulted in a significant increase in fruit size compared to unsprayed control trees (Fig. 12).

**Fig. 13:**
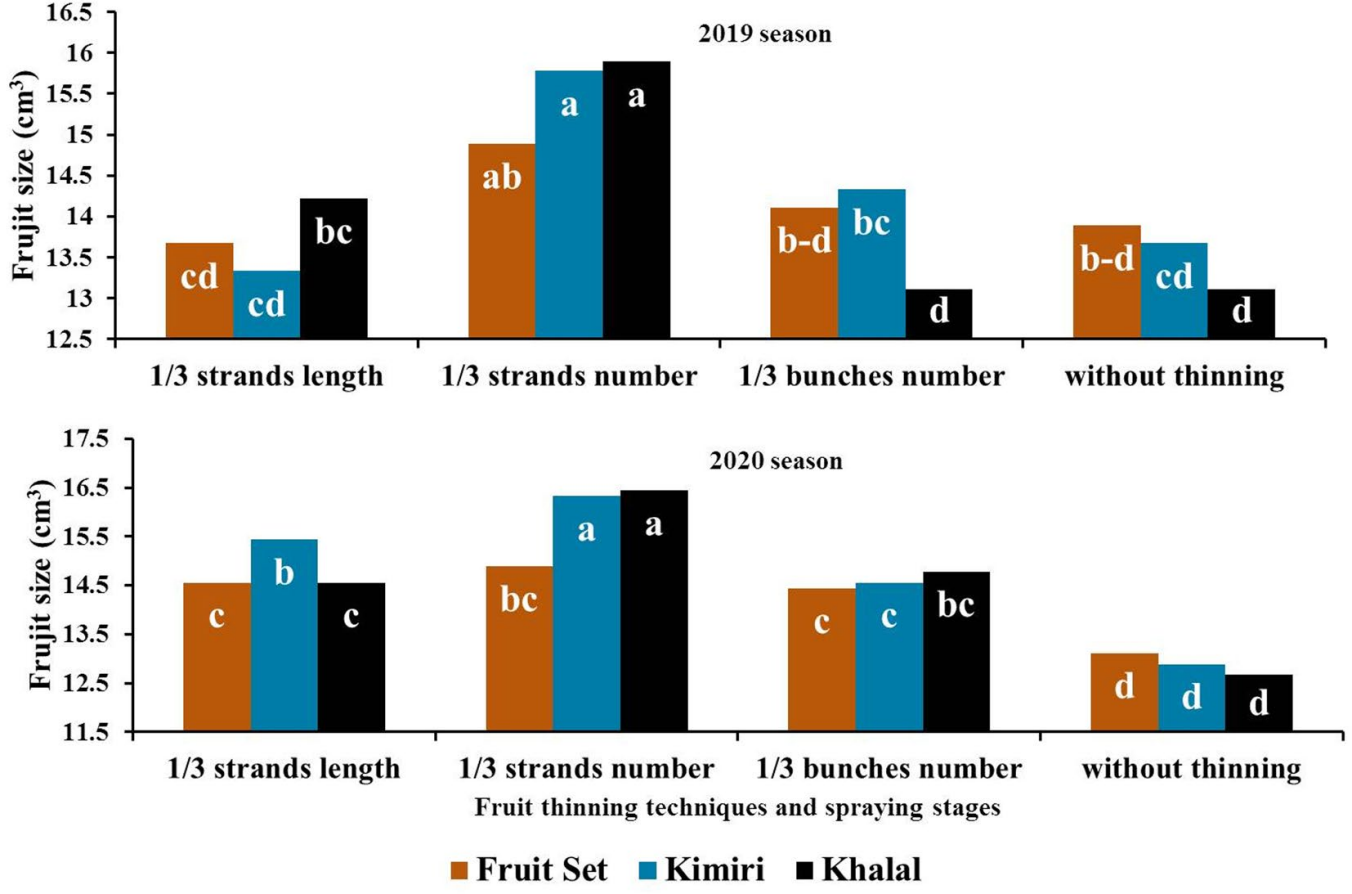
Effect of different thinning techniques (1/3 strands length, 1/3 strands number, 1/3 bunches number and control) combined with spraying stages (fruit set, Kimri and Khalal ) on fruit size of Medjool date palm during 2019 and 2020 seasons.

Regarding the impact of the spraying stage, generally spraying at Kimri stage, when combined with removing 1/3 strands number increased fruit size with significant values in the second season (Fig. 13).

Regarding the triple interaction, removing 1/3 strands number combined with spraying CaB even at Kimri or Khalal stages stage increased fruit size (Table 6). Similarly, removing 1/3 strands number combined with spraying KP at Kimri stage succeeded in increase fruit size.

**Table 6:** Effect of different thinning methods and spraying substance at different fruit growth stages on Medjool fruit size (cm3).

Spraying stages	Spraying substances	Thinning methods by removing					
		1/3 strands length	1/3 strands number		1/3 bunches number		Without thinning
**Fruit size (cm** ^3^ **) in 2019 season**							
Fruit set	Control	11.33 k	13.67 e-i		12.33 h-k		12.33 h-k
	0.25% CaB	15.33 c-e	15.67 b-d		15.67 b-d		14.67c-g
	0.2%KP	14.33 d-g	15.33 c-e		14.33 d-g		14.67c-g
Kimiri	Control	12.00 i-k	13.67 e-i		13.33 f-j		11.67jk
	0.25% CaB	11.67 jk	17.33 ab		15.67 b-d		14.33d-g
	0.2%KP	16.33 a-c	16.33 a-c		14.00 d-h		15.00c-f
Khalal	Control	11.33 k	14.00 d-h		12.33 h-k		11.67 jk
	0.25% CaB	16.33 a-c	18.00 a		11.67 jk		14.67c-g
	0.2%KP	15.00 c-f	15.67 b-d		15.33 c-e		13.00 g-k
**Fruit size (cm** ^3^ **) in 2020 season**							
Fruit set	Control	13.00 h-j	13.33 g-i		12.67 ij		11.67 j
	0.25% CaB	14.67 d-g	16.00 b-d		15.33 c-f		13.33 g-i
	0.2%KP	16.00 b-d	15.33 c-f		15.33 c-f		14.33e-h
Kimiri	Control	13.67 g-i	13.33 g-i		13.00 h-j		11.67 j
	0.25% CaB	16.33 bc	18.33 a		15.33 c-f		12.67 ij
	0.2%KP	16.33 bc	17.33 ab		15.33 c-f		14.33e-h
Khalal	Control	13.00 h-j	14.00 f-i		13.33 g-i		11.67 j
	0.25% CaB	15.33 c-f	18.00 a		15.67 c-e		13.00 h-j
	0.2%KP	15.33 c-f	17.33 ab		15.33 c-f		13.33 g-i

The values following the same letter in each season do not differ significantly at a probability level of 0.05. CaB = Calcium boron, KP = Potassium phosphite.

It can be concluded that removing 1/3 strands number improved all physical parameters of Medjool fruit. Spraying at the Kimri stage also had a similar positive impact. Additionally, removing 1/3 strands number combined with CaB at either the Kimri or Khalal stage further enhanced Medjool fruit’s physical properties. Our findings were aligned with El-Motaium et al.^31^ who observed an increase in mango fruit volume as a result of foliar spraying of 250 ppm B + 500 ppm Ca. Also, spraying Medjool date palms with 1% CaNO_3_ or 0.5% CaCl_2_ significantly increased fruit diameter and length^34^. Since K regulates cytokinin levels, which regulates the cell division process^43,44^.

### Fruit chemical characteristics

#### Fruit TSS (%)

**Fig. 14:**
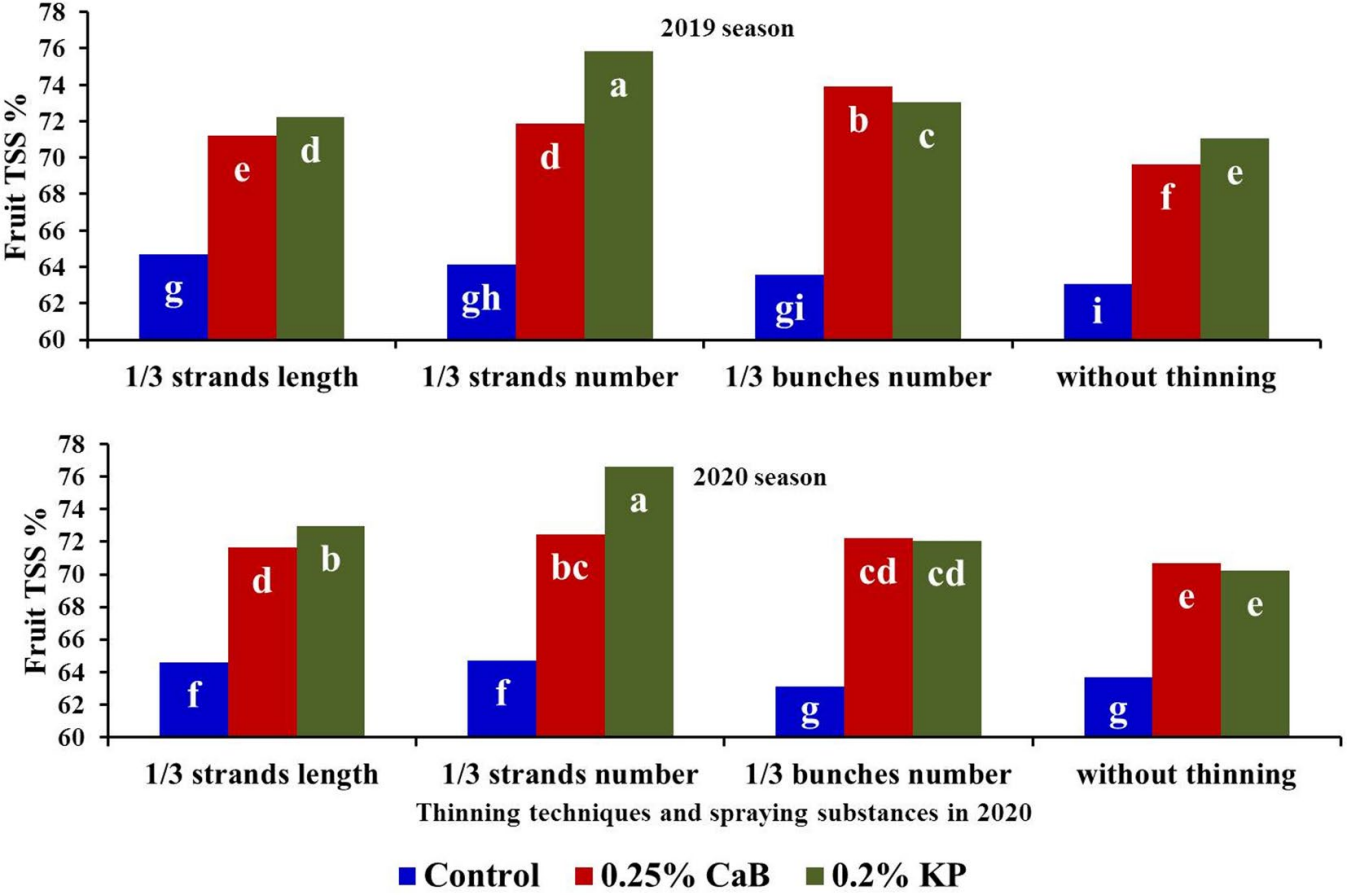
Effect of different thinning techniques (1/3 strands length, 1/3 strands number, 1/3 bunches number and control) combined with spraying substances (0.25% CaB, 0.2% KP and control) on fruit TSS % of Medjool date palm during 2019 and 2020 seasons.

The results revealed that removing 1/3 strands number resulted in the highest fruit TSS content, whereas the un-thinned control treatment had the lowest values in both seasons (Fig. 14). Concerning the effect of spraying the substance, it can be noticed that both KP and CaB significantly increased fruit TSS % compared to the non-sprayed control with all thinning treatments. Moreover, spraying KP recorded the highest significant fruit TSS content followed by CaB when combined with removing either 1/3 strands number or 1/3 strands length, while the non-sprayed control recorded the lowest significant values (Fig. 14).

**Fig. 15:**
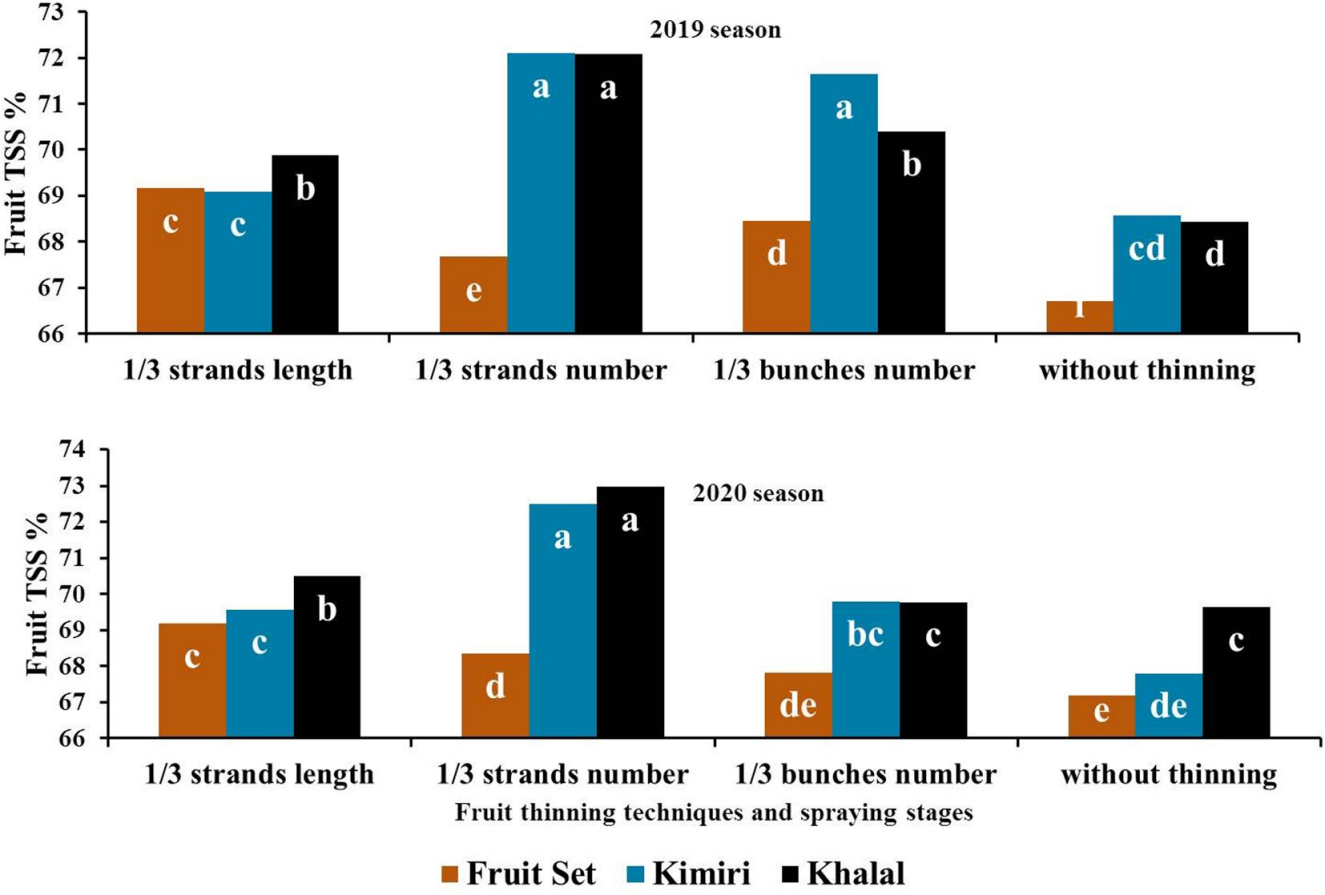
Effect of different thinning techniques (1/3 strands length, 1/3 strands number, 1/3 bunches number and control) combined with spraying stages (fruit set, Kimri and Khalal ) on fruit TSS % of Medjool date palm during the 2019 and 2020 seasons.

Regarding the effect of the spraying stage, generally spraying at Khalal and Kimri stages (the later fruit growth stages) increased fruit TSS% compared to spraying after fruit set stage, especially when combined with removing 1/3 strands number (Fig. 15).

Concerning the triple interaction, removing 1/3 strands number combined with spraying KP at the Kimri or Khalal stage or spraying by CaB at Khalal stage significantly increased TSS fruit content (Table 7).

**Table 7:** Effect of different thinning methods and spraying substances at different fruit growth stages on Medjool fruit TSS content %.

Spraying stages	Spraying substances	Thinning methods by removing			
		1/3 strands length	1/3 strands number	1/3 bunches number	Without thinning
**Fruit TSS (Brix) in 2019 season**					
Fruit set	Control	64.37 mn	64.20 n	63.90 no	64.37mn
	0.25% CaB	71.30 ij	64.50 mn	72.10 g-i	67.10 l
	0.2%KP	71.80 hi	74.30 de	69.37 k	68.70k
Kimiri	Control	64.37 mn	64.20 n	63.90 no	62.40p
	0.25% CaB	69.40 k	75.10 cd	75.40 bc	70.60j
	0.2%KP	73.50 ef	77.00 a	75.63 bc	72.70f-h
Khalal	Control	65.33 m	64.00 n	62.90 op	62.40p
	0.25% CaB	72.90 fg	76.00 ab	74.20 de	71.23 ij
	0.2%KP	71.40 ij	76.20 ab	74.10 de	71.70 hi
**Fruit TSS (Brix) in 2020 season**					
Fruit set	Control	64.20 m	64.20 m	62.40 n	62.73 n
	0.25% CaB	71.50 e-g	65.50 j-l	71.57 e-g	69.77 hi
	0.2%KP	71.83 d-g	75.33 b	69.50 i	69.03 i
Kimiri	Control	65.30 j-m	64.47 k-m	62.63 n	62.33 n
	0.25% CaB	69.37 i	75.40 b	72.70 de	70.93gh
	0.2%KP	74.03 c	77.60 a	74.03 c	70.13hi
Khalal	Control	64.33 k-m	65.53 jk	64.27 lm	66.00 j
	0.25% CaB	74.07 c	76.50 ab	72.33 d-f	71.40 fg
	0.2%KP	73.07 cd	76.87 a	72.70 de	71.53e-g

The values following the same letter in each season do not differ significantly at a probability level of 0.05. CaB = calcium boron, KP = potassium phosphite.

Ca improves fruit TSS^23,28^. B increases fruit sugar content through increasing the transport of carbohydrates, which leads to increased TSS and sugars in fruit as well as increased fruit dry matter^33^. These findings align with Madani et al.^7^, who found that thinning by removing one-third of the terminal tips of central strands in early Kimri led to an increase in TSS content in Piyarom date palm compared to thinning by removing one-third of total strands from terminal tips during pollination and the un-thinned control. Also, El-Assar and Refaat^42^ reported that thinning of Sewy bunches by 20 and 40% increased TSS of Sewy date fruits. Moreover, Al-hajjaj et al.^40^ found that 800ppm K gave the highest TSS of Medjool date fruits. Also, Al-hajjaj and Ayad^41^ found that spraying with boron at 400 and 800 ppm resulted in an increasing TSS content of Medjool fruit. Our findings were agreed with El-Motaium et al.^31^ who observed an increase in mango fruit TSS content due to foliar spraying of 250 ppm B + 500 ppm Ca. Also, spraying date palms 1% CaNO_3_ or 0.5% CaCl_2_ significantly improved fruit TSS of Medjool dates^34^. Furthermore, potassium citrate of 0.5% incorporated with 0.2% methionine improved fruit quality (TSS and total sugars) of Balady mandarin trees^17^ (Tables 8, 9).

**Table 8:** Effect of different thinning methods and spraying substances at different fruit growth stages on Medjool fruit moisture content%.

Spraying stages	Spraying substances	Thinning methods by removing			
		1/3 strands length	1/3 strands number	1/3 bunches number	Without thinning
**Fruit moisture content% in 2019 season**					
Fruit set	Control	20.60 e-h	21.50 c-e	20.20 f-i	20.27e-h
	0.25% CaB	21.50 c-e	19.80 hi	25.33 a	24.20 ab
	0.2%KP	22.00 cd	20.00 g-i	20.30 f-i	19.87 g-i
Kimiri	Control	20.00 g-i	24.70 ab	22.20 c	19.60 hi
	0.25% CaB	20.40 e-h	19.70 hi	20.37 e-i	19.87 g-i
	0.2%KP	21.00 d-g	19.90 g-i	21.23 c-f	19.20 i
Khalal	Control	20.00 g-i	24.70 ab	22.20 c	19.60 h-i
	0.25% CaB	20.33 f-i	19.80 hi	24.80 ab	20.10 f-i
	0.2%KP	21.20 c-f	20.70 e-h	24.80 ab	24.00 b
**Fruit moisture content% in 2020 season**					
Fruit set	Control	20.80 hi	21.50 f-i	22.30 b-h	21.50 f-i
	0.25% CaB	22.40 b-h	21.90d-i	24.10 ab	24.30a
	0.2%KP	23.20 a-f	23.90 a-c	23.80 a-c	21.77e-i
Kimiri	Control	21.80 d-i	23.90 a-c	20.30 i	22.47b-h
	0.25% CaB	21.60 f-i	20.80 d-i	21.80 d-i	22.13c-h
	0.2%KP	22.90 a-g	22.40 b-h	23.60 a-d	21.50 f-i
Khalal	Control	20.80 hi	21.50 f-i	22.30 b-h	21.50 f-i
	0.25% CaB	21.23 g-i	21.70 e-i	23.90 a-c	22.40b-h
	0.2%KP	22.10 c-i	23.50 a-e	24.10 ab	22.90a-g

The values following the same letter in each season do not differ significantly at a probability level of 0.05. CaB = calcium boron, KP = potassium phosphite.

**Table 9:** Effect of different thinning methods and spraying substances at different fruit growth stages on Medjool fruit titratable acidity %.

Spraying stages	Spraying substances	Thinning methods by removing			
		1/3 strands length	1/3 strands number	1/3 bunches number	Without thinning
**Fruit titratable acidity% in 2019 season**					
Fruit set	Control	0.13 c	0.12 c	0.15 c	0.20 a-c
	0.25% CaB	0.10 c	0.08 c	0.06 c	0.19 a-c
	0.2%KP	0.10 c	0.09 c	0.33 ab	0.15 c
Kimiri	Control	0.13 c	0.15 c	0.15 c	0.20 a-c
	0.25% CaB	0.10 c	0.07 c	0.36 a	0.18 bc
	0.2%KP	0.10 c	0.09 c	0.08 c	0.17 bc
Khalal	Control	0.13 c	0.12 c	0.20 a-c	0.20 a-c
	0.25% CaB	0.10 c	0.09 c	0.08 c	0.17 bc
	0.2%KP	0.12 c	0.10 c	0.07 c	0.16 bc
**Fruit titratable acidity% in 2020 season**					
Fruit set	Control	0.15 b-d	0.19 a-d	0.15 b-d	0.20 a-d
	0.25% CaB	0.09 cd	0.09 cd	0.09 cd	0.14 b-d
	0.2%KP	0.09 cd	0.09 cd	0.28 a-c	0.14 b-d
Kimiri	Control	0.13 b-d	0.15b-d	0.15 b-d	0.2 a-d
	0.25% CaB	0.10 cd	0.08 d	0.36 a	0.12 cd
	0.2%KP	0.10 cd	0.08 d	0.08 d	0.1 cd
Khalal	Control	0.12 b-d	0.12 cd	0.19 a-d	0.2 a-c
	0.25% CaB	0.09 cd	0.09 cd	0.32 ab	0.17 a-d
	0.2%KP	0.09 cd	0.10 cd	0.09 cd	0.15 b-d

The values following the same letter in each season do not differ significantly at a probability level of 0.05. CaB = calcium boron, KP = potassium phosphite.

#### Fruit moisture content (%)

**Fig. 16:**
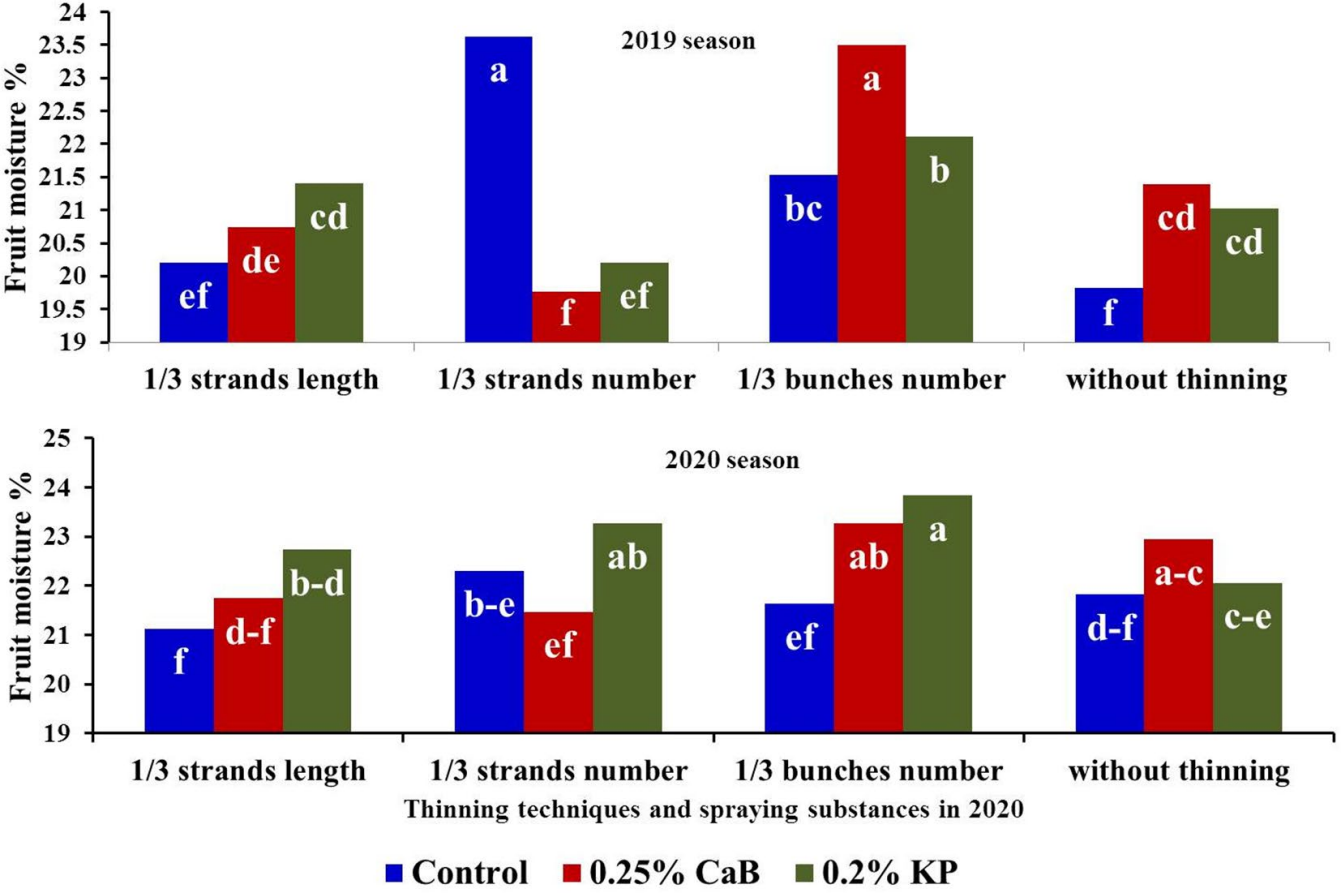
Effect of different thinning techniques (1/3 strands length, 1/3 strands number, 1/3 bunches number and control) combined with spraying substances (0.25% CaB, 0.2% KP and control) on fruit moisture % of Medjool date palm during 2019 and 2020 seasons.

The results indicated that, removing 1/3 bunches number significantly increased fruit moisture content compared the other thinning treatments, while the lowest value was recorded by removing 1/3 strands length. Regarding the effect of spraying substances, spraying KP increased fruit moisture content compared with spraying CaB in the second season in thinned date palm trees(Fig. 16).

**Fig. 17:**
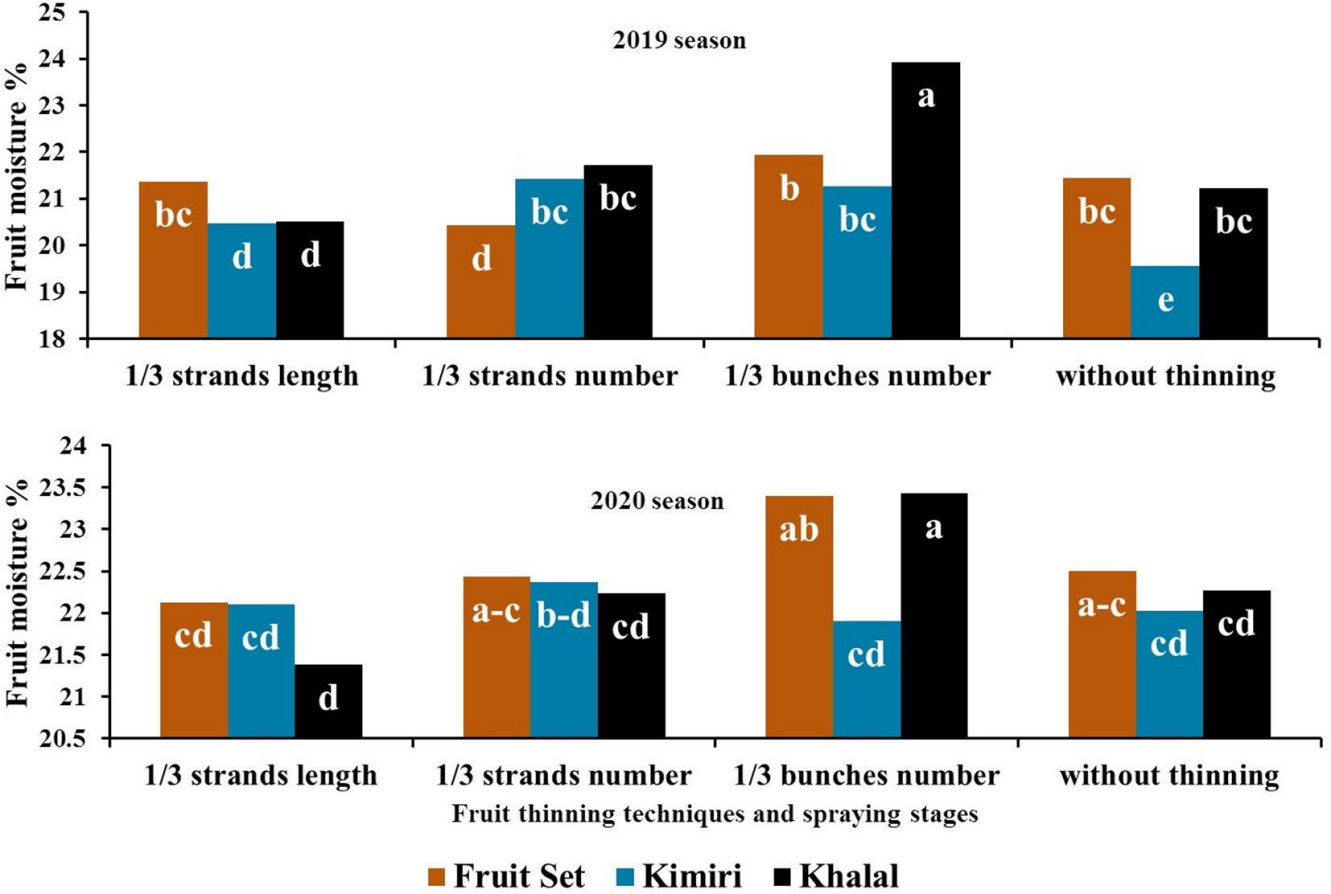
Effect of different thinning techniques (1/3 strands length, 1/3 strands number, 1/3 bunches number and control) combined with spraying stages (fruit set, Kimri and Khalal ) on fruit moisture % of Medjool date palm during 2019 and 2020 seasons.

Concerning the effect of the spraying stage, spraying at Khalal stage combined with removing 1/3 bunches number significantly increased fruit moisture content compared to spraying at different stages combined with removing 1/3 strands length (Fig. 17). This means that, later spraying increases the moisture content in the fruit which may result from delaying repining stage and stimulating fruit growth. Removing 1/3 strands length decreased moisture content than removing 1/3 bunches number of removing 1/3 strands number especially during Kimri and Khalal stages.

Regarding the triple interaction, removing1/3 bunches number combined with CaB spraying after fruit set or combined with KP spraying at Khalal stage significantly increased the fruit moisture content compared to the other interaction. These results were agreement with Soliman et al.^47^ who showed that bunches thinning of Khalas date palm by a rate of 30% increased fruit moisture content. Also, Al-hajjaj and Ayad^41^ found that spraying B at 1200 and 1600ppm increased Medjool fruit moisture content. Foliar application of 1500ppm B at the Khalal and rutab stages increased dry matter and TSS of Halawi and Sayer date fruits^34^. Furthermore, Sobeih^35^ reported that, spraying Al-Sayer date palm trees with 300ppm B significantly increased fruit water content.

#### Fruit acidity content

**Fig. 18:**
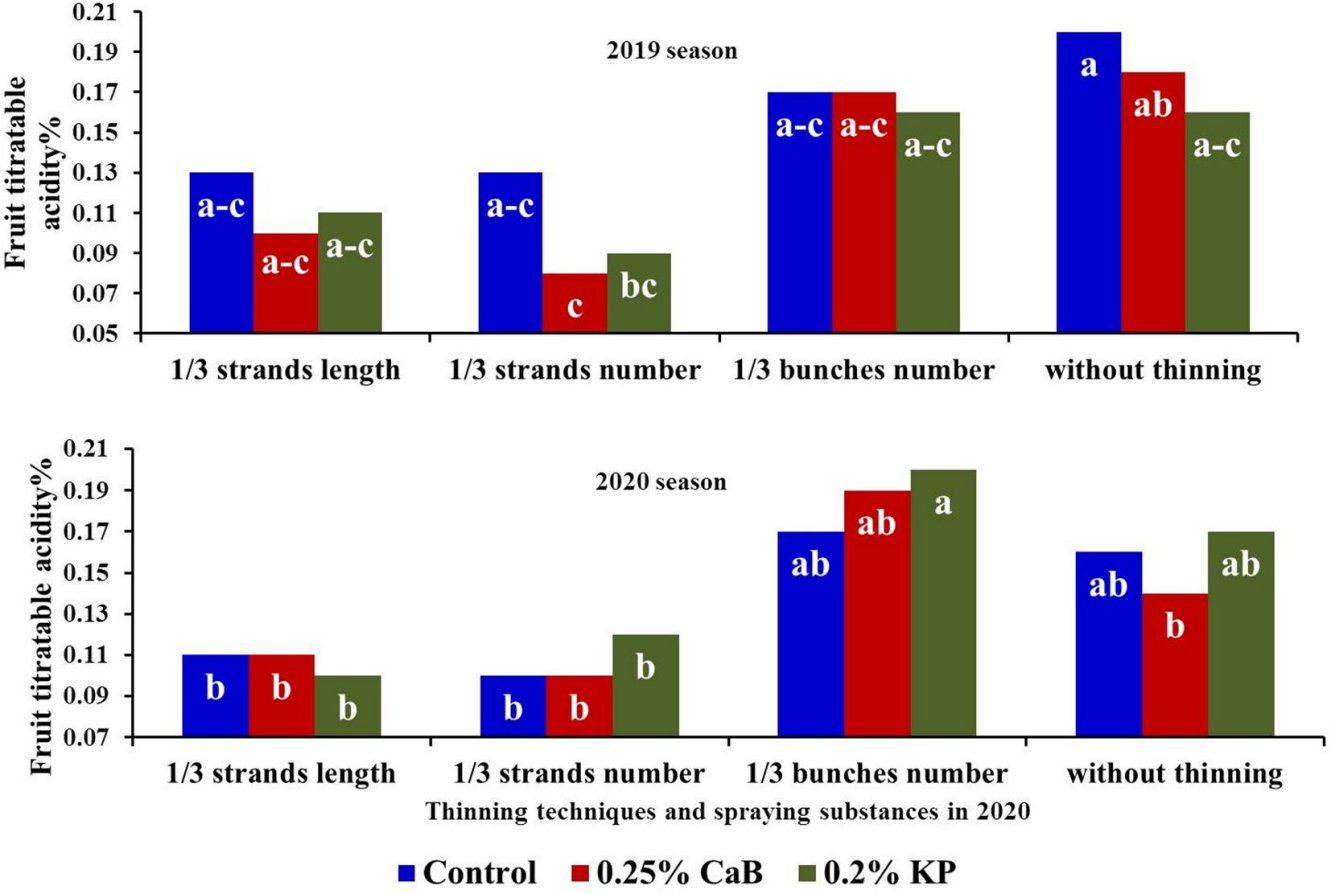
Effect of different thinning techniques (1/3 strands length, 1/3 strands number, 1/3 bunches number and control) combined with spraying substances (0.25% CaB, 0.2% KP and control) on fruit titratable acidity% of Medjool date palm during 2019 and 2020 seasons.

Removing 1/3 strands number recorded the lowest significant fruit acidity content, followed by removing 1/3 strands length, while un-thinned control and removing 1/3 bunches number recorded the highest fruit acidity content (Fig. 18).

**Fig. 19:**
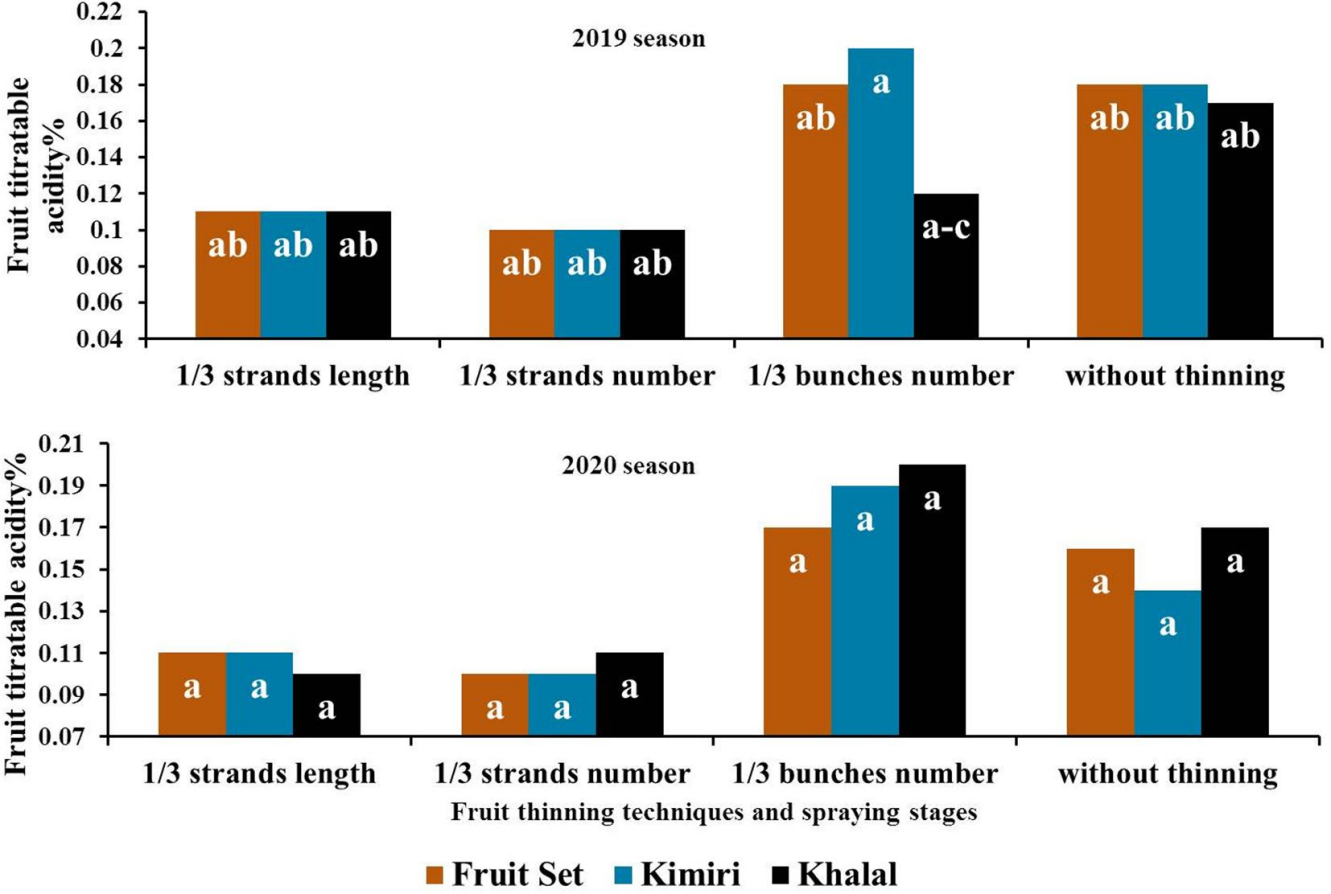
Effect of different thinning techniques (1/3 strands length, 1/3 strands number, 1/3 bunches number and control) combined with spraying stages (fruit set, Kimri and Khalal ) on fruit titratable acidity % of Medjool date palm during 2019 and 2020 seasons.

Regarding the effect of spraying stage and spraying substance, the data showed no significant differences between the treatments in fruit titratable acidity content (Fig. 19). A slight increase was observed in removing 1/3 bunches number and un-thinned date palm trees at different fruit stages.

Regarding the triple interaction, the results indicated that both removing 1/3 bunches number and 1/3 strands number, combined with KP spraying at Kimri stage, reduced fruit acidity content. These findings were consistent with those of Soliman et al.^47^ who found that bunch thinning reduced fruit acidity of Khalas dates. Also, spraying Medjool date palm with 1% CaNO_3_ or 0.5% CaCl_2_ reduced fruit acidity^34^. El-Motaium et al.^31^ found a reduction in mango fruit acidity as a result of foliar spraying of 250 ppm B + 500 ppm Ca.

#### Total sugars content (%)

**Fig. 20:**
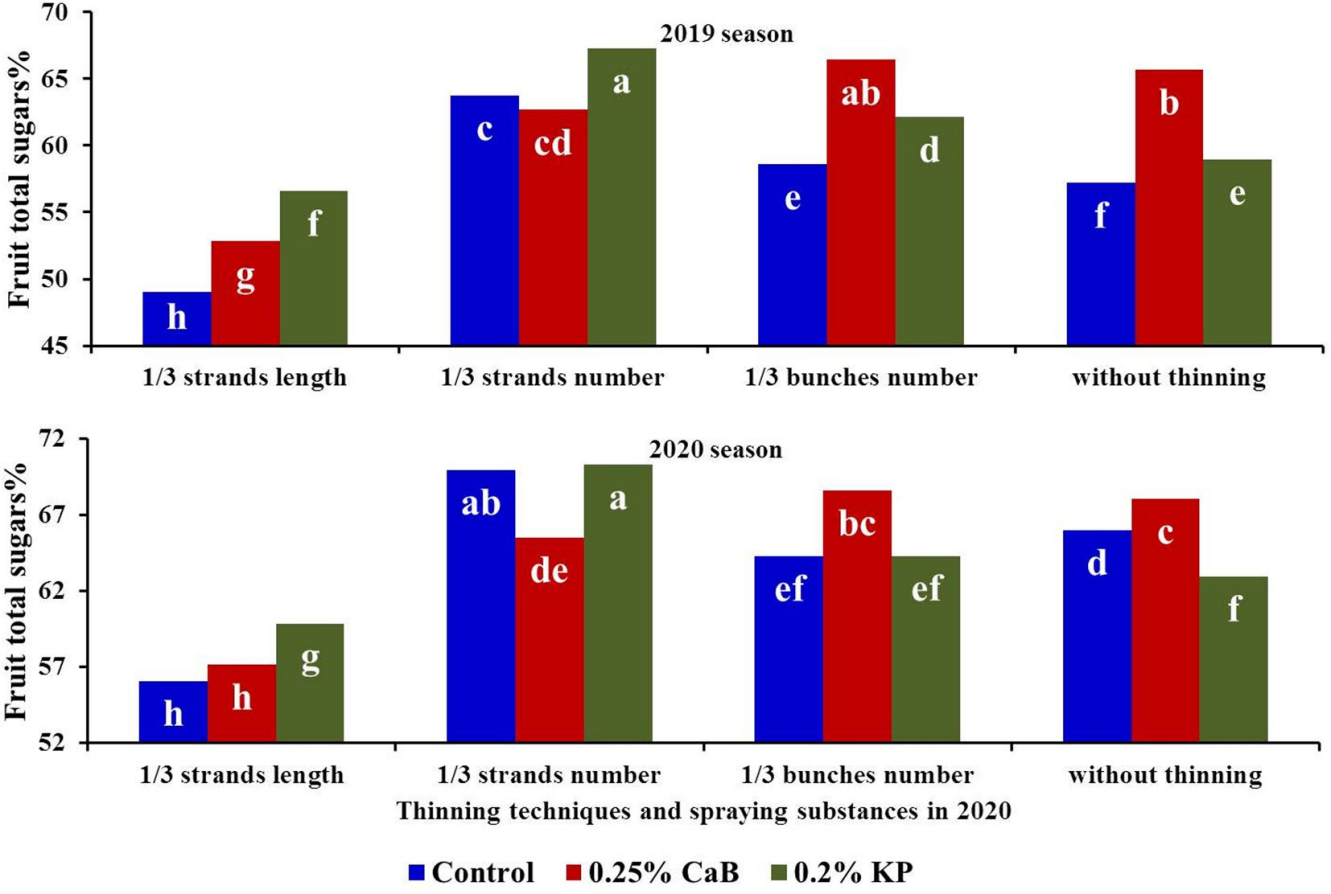
Effect of different thinning techniques (1/3 strands length, 1/3 strands number, 1/3 bunches number and control) combined with spraying substances (0.25% CaB, 0.2% KP and control) on fruit total sugars of Medjool date palm during 2019 and 2020 seasons.

Removing 1/3 strands number recorded the highest percentage of fruit total sugars content, followed by removing 1/3 bunches number, while removing 1/3 strands length recorded the lowest values (Fig. 20). Also, spraying CaB showed higher significant sugars compared to spraying KP when combined with both removing 1/3 strands length and removing 1/3 strands number in both seasons (Fig. 20).

**Fig. 21:**
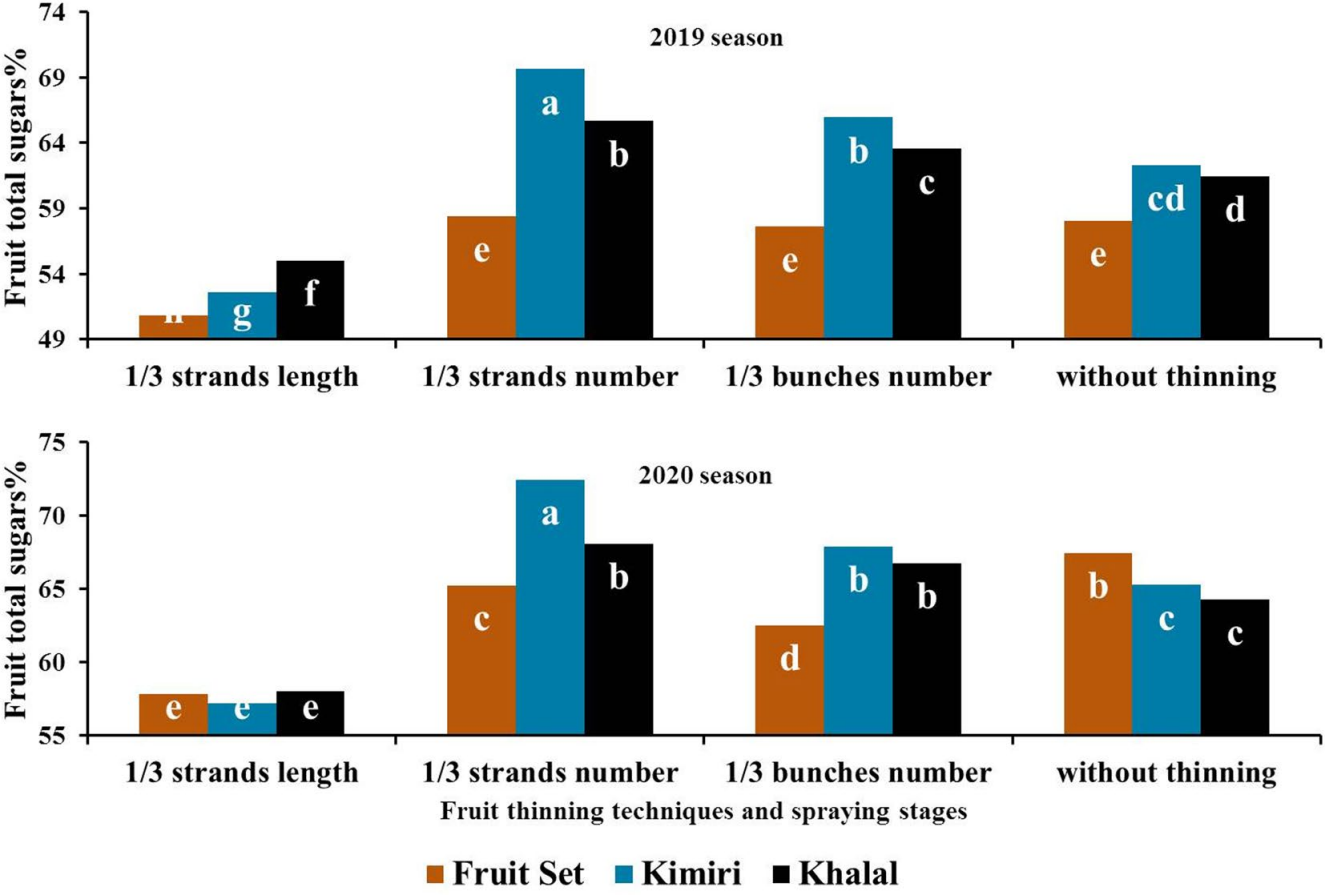
Effect of different thinning techniques (1/3 strands length, 1/3 strands number, 1/3 bunches number and control) combined with spraying stages (fruit set, Kimri and Khalal ) on fruit total sugars % of Medjool date palm during 2019 and 2020 seasons.

Concerning the effect of spraying stage, the data showed that, generally spraying at Kimri stage recorded higher total sugar fruit content followed by Khalal, while early spraying at fruit set stage recorded the lowest sugars content in both seasons (Fig. 21).

Regarding the triple interaction, removing 1/3 strands number combined with either KP spraying at Kimri stage or removing 1/3 bunches number combined with CaB spraying at Kimri stage recorded the highest fruit reducing sugar content compared with other interactions in the study (Table 10).

**Table 10:** Effect of different thinning methods and spraying substances at different fruit growth stages on Medjool fruit total sugars %.

Spraying stages	Spraying substances	Thinning methods by removing			
		1/3 strands length	1/3 strands number	1/3 bunches number	Without thinning
**Fruit total sugars% in 2019 season**					
Fruit set	Control	46.00 o	59.33 hi	56.47 j-l	51.73 n
	0.25% CaB	50.80 n	54.37 lm	56.80 jk	67.77de
	0.2%KP	55.60 kl	61.53 f-h	59.60 hi	54.7 km
Kimiri	Control	55.00 kl	72.60 bc	62.80 f	68.10de
	0.25% CaB	41.17 p	62.03 fg	76.53 a	56.77jk
	0.2%KP	61.60 f-h	74.23 b	58.50 ij	62.03 fg
Khalal	Control	46.00 o	59.33 hi	56.47 j-l	51.73 n
	0.25% CaB	66.53 de	71.57 c	65.90 e	72.53bc
	0.2%KP	52.53 mn	66.10 de	68.30 d	60.13gi
**Fruit total sugars% in 2020 season**					
Fruit set	Control	59.07 m-o	73.87 bc	66.00 hi	70.43 df
	0.25% CaB	54.80 q	58.33 n-p	60.90 lm	71.17 de
	0.2%KP	59.60 mn	63.57 i-k	60.73 l-n	60.80 lm
Kimiri	Control	59.07 m-o	73.87 bc	66.00 hi	70.43d-f
	0.25% CaB	49.07 r	64.57 ij	75.60 b	60.87 lm
	0.2%KP	63.40 jk	78.93 a	62.10j-l	64.53 ik
Khalal	Control	50.00 r	62.17 j-l	60.87 lm	57.13oq
	0.25% CaB	67.57 gh	73.67 bc	69.33 e-g	72.13 cd
	0.2%KP	56.53 h	68.47 fg	70.10 d-f	63.50 jk

The values following the same letter in each season do not differ significantly at a probability level of 0.05. CaB = Calcium boron, KP = Potassium phosphite.

#### Reducing sugar content (%)

**Fig. 22:**
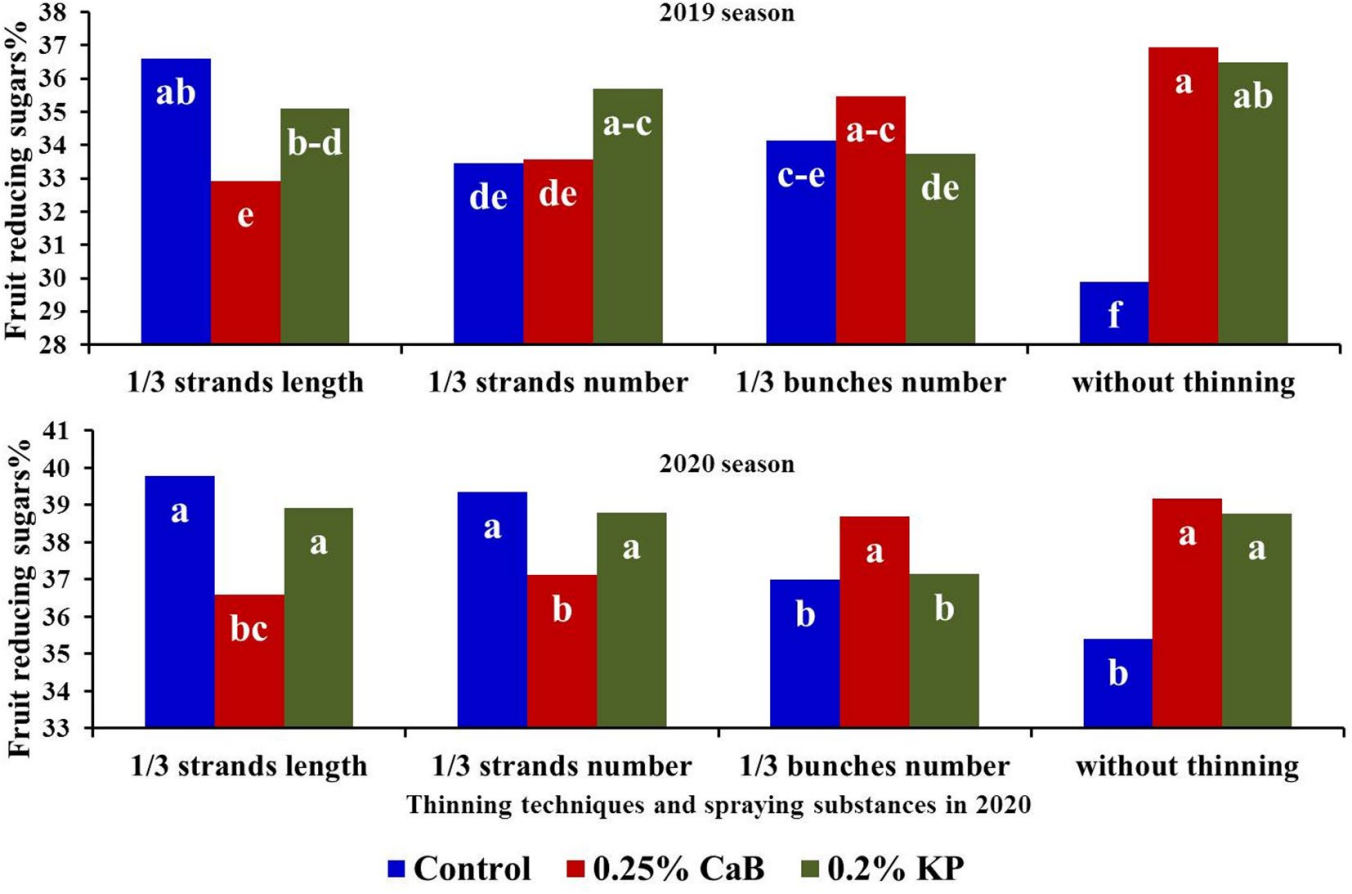
Effect of different thinning techniques (1/3 strands length, 1/3 strands number, 1/3 bunches number and control) combined with spraying substances (0.25% CaB, 0.2% KP and control) on fruit reducing sugars % of Medjool date palm during 2019 and 2020 seasons.

The results indicated that un-thinned palm, removing 1/3 strands length and removing 1/3 strands number method led to a significant increase in fruit reducing sugar content compared to the other thinning treatments in the second season (Fig. 22). Regarding the effect of spraying the substance, it can be noticed that spraying KP and CaB led to a significant increase in fruit reducing sugars content in the first season. In un-thinned palm trees, spraying substances resulted in a high significant increase in reducing sugar (Fig. 22). Also, removing 1/3 bunches number combined with spraying KP gave high increase in reducing sugars which become significant in the second season compared to spraying with CaB and control (water sprayed only).

**Fig. 23:**
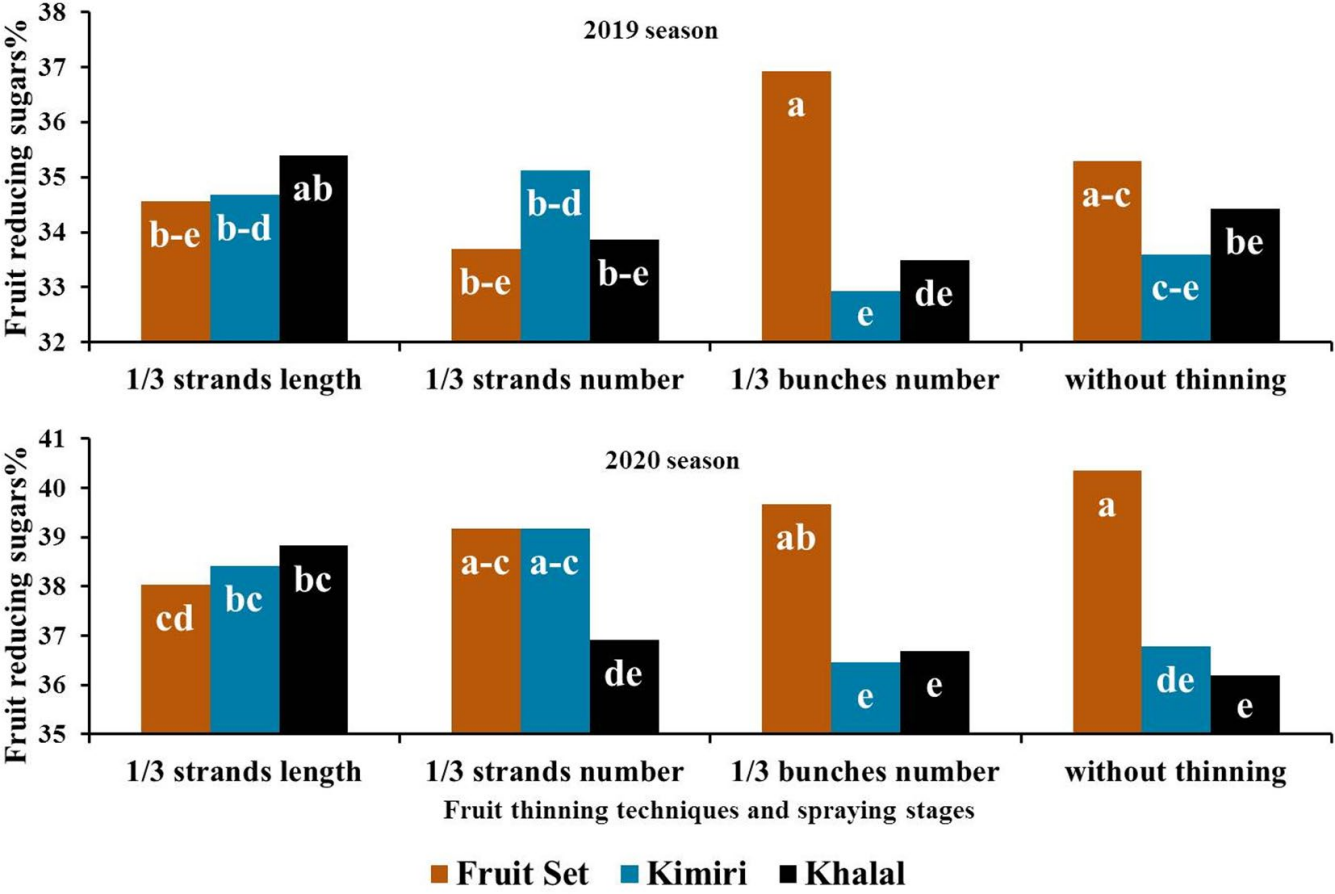
Effect of different thinning techniques (1/3 strands length, 1/3 strands number, 1/3 bunches number and control) combined with spraying stages (fruit set, Kimri and Khalal ) on fruit reducing sugars % of Medjool date palm during 2019 and 2020 seasons.

Concerning the effect of the spraying stage, the results indicated that spraying after fruit set stage significantly increased reducing sugar compared to spraying at Kimri and Khalal stages, particularly when combined with removing 1/3 bunches number or un-thinned palms (Fig. 23).

Regarding the triple interaction, the results showed that removing 1/3 bunches number thinning method combined with CaB spraying after the fruit set recorded the highest fruit reducing sugar content (Table 11).

**Table 11:** Effect of different thinning methods and spraying substances at different fruit growth stages on Medjool fruit reducing sugar %.

Spraying stages	Spraying substances	Thinning methods by removing			
		1/3 strands length	1/3 strands number	1/3 bunches number	Without thinning
**Fruit reducing sugars% in 2019 season**					
Fruit set	Control	37.07 b-d	31.83 i-l	34.30 d-i	27.83 m
	0.25% CaB	31.93 i-l	35.27 d-h	41.10 a	39.37 ab
	0.2%KP	34.67 d-i	34.00 e-j	35.40 d-g	38.67 ab
Kimiri	Control	35.70 c-g	36.70 b-e	33.80 e-j	34.00e-i
	0.25% CaB	31.27 j-l	29.50 lm	32.93 g-k	30.63 km
	0.2%KP	37.07 b-d	39.20 ab	32.07 i-l	36.20c-f
Khalal	Control	37.07 b-d	31.83 i-l	34.30 d-i	27.83 m
	0.25% CaB	35.60 d-g	35.90 c-g	32.40 h-l	40.83 a
	0.2%KP	33.53 f-k	33.83 e-j	33.77 e-j	34.63d-i
**Fruit reducing sugars% in 2020 season**					
Fruit set	Control	39.47 e-g	41.53 a-d	36.93 h-m	37.50 gk
	0.25% CaB	36.10 k-m	38.73 e-j	43.17 a	42.83 ab
	0.2%KP	38.53 f-j	37.23 g-l	38.90 e-i	40.70c-f
Kimiri	Control	39.47 d-g	41.53 a-d	36.93 h-m	37.50 gk
	0.25% CaB	34.90 mn	33.53 n	36.70 i-m	33.80 n
	0.2%KP	40.90 a-e	42.43 a-c	35.73 k-n	39.03 eh
Khalal	Control	40.37 c-f	34.97 l-n	37.07 h-m	31.20 o
	0.25% CaB	38.77 e-j	39.07 e-h	36.20 k-m	40.83 be
	0.2%KP	37.33 g-k	36.70 i-m	36.80 h-m	36.57jm

The values following the same letter in each season do not differ significantly at a probability level of 0.05. CaB = calcium boron, KP =  potassium phosphite.

#### Non-reducing sugar content (%)

**Fig. 24:**
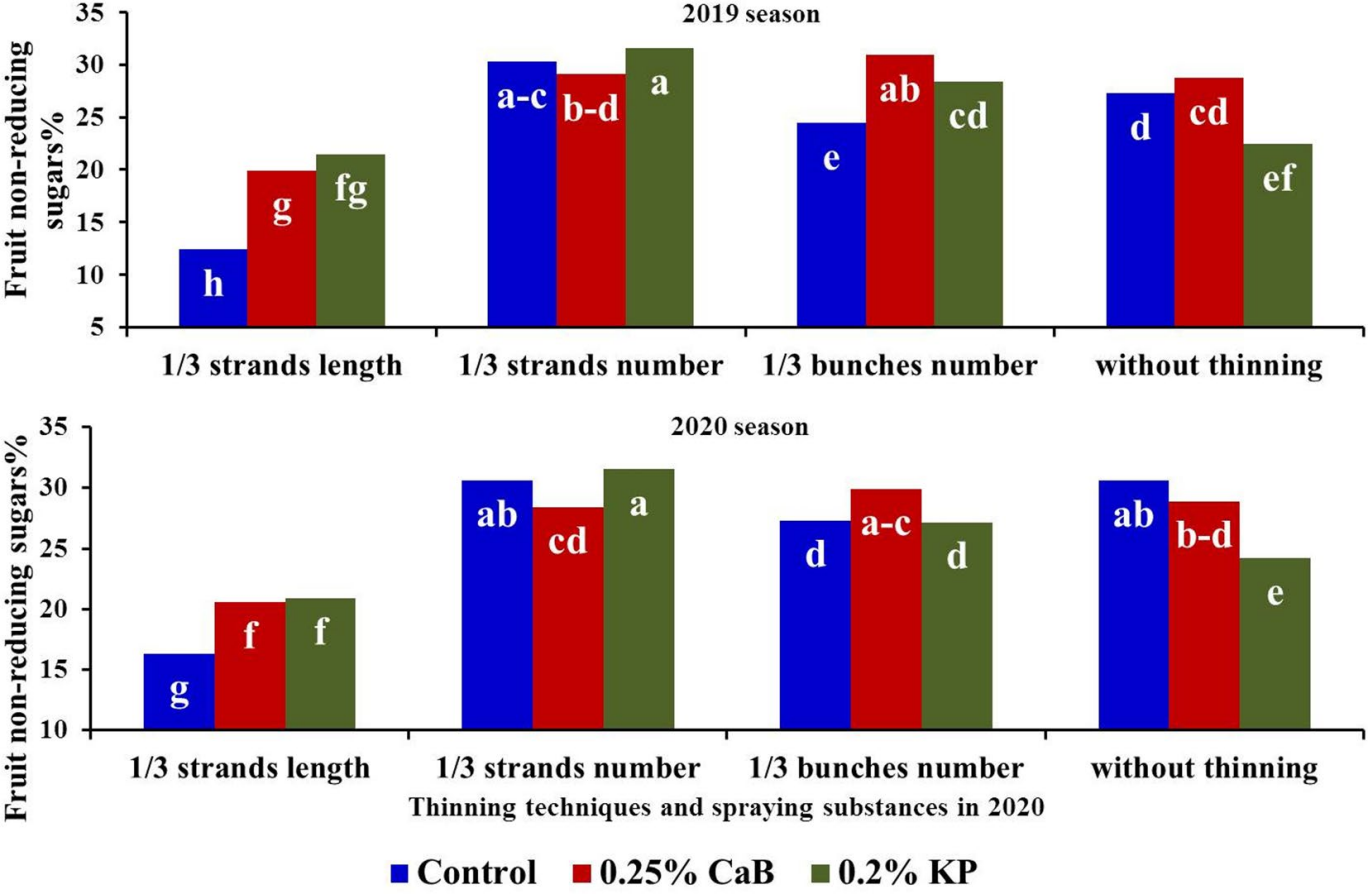
Effect of different thinning techniques (1/3 strands length, 1/3 strands number, 1/3 bunches number and control) combined with spraying substances (0.25% CaB, 0.2% KP and control) on fruit weight of Medjool date palm during 2019 and 2020 seasons.

The results indicated that removing 1/3 strands number followed by removing 1/3 bunches number led to a significant increase in fruit non-reducing sugars content compared to the other thinning treatments, especially when combined with KP spraying (Fig. 24). As for the effect of spraying substances, it can be noticed that, CaB spraying significantly increased fruit non-reducing sugar content compared to KP in the both seasons in all thinning treatments except for removing 1/3 strands length (Fig. 24).

**Fig. 25:**
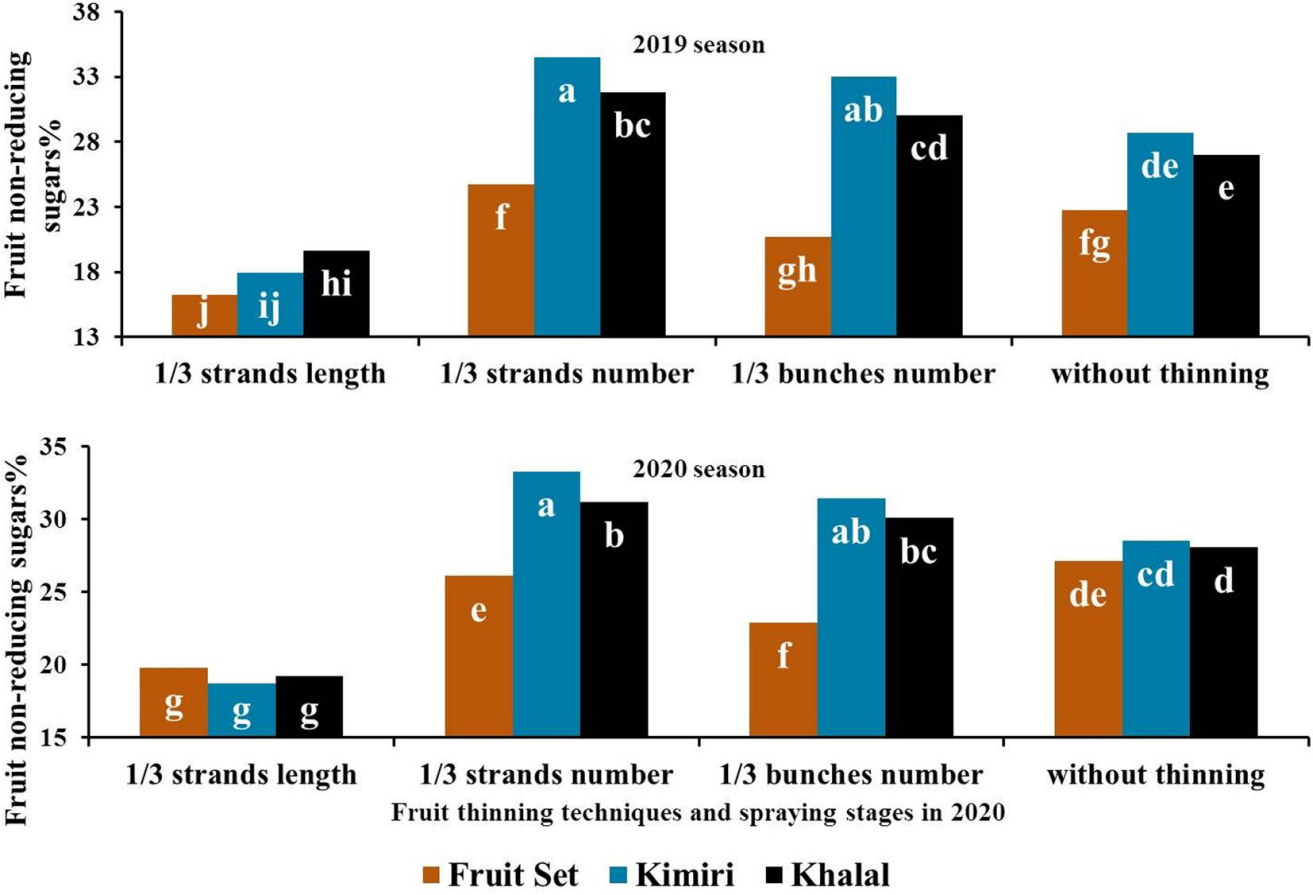
Effect of different thinning techniques (1/3 strands length, 1/3 strands number, 1/3 bunches number and control) combined with spraying stages (fruit set, Kimri and Khalal ) on fruit non-reducing sugars % of Medjool date palm during 2019 and 2020 seasons.

Regarding the effect of spraying stage, the results showed that, spraying at Kimri stage increased non-reducing sugar compared to spraying at Khalal stage, whereas spraying after fruit set stage gave the lowest values. This indicates that early spraying had little effect on accumulation of non-reducing sugars compared to later spraying (Fig. 25).

With regard to the triple interaction, the results showed that removing 1/3 bunches number per date palm combined with CaB spraying at Kimri stage recorded the highest significant fruit non-reducing sugar content (Table 12).

**Table 12:** Effect of different thinning methods and spraying substances at different fruit growth stages on Medjool fruit non-reducing sugar %.

Spraying stages	Spraying substances	Thinning methods by removing			
		1/3 strands length	1/3 strands number	1/3 bunches number	Without thinning
**Fruit non-reducing sugars% in 2019 season**					
Fruit set	Control	8.93 n	27.50 g-i	22.17 i-l	23.9 i-l
	0.25% CaB	18.87 lm	19.10 lm	15.70 m	28.4f-h
	0.2%KP	20.93 kl	27.53 g-i	24.20 i-k	16.0 m
Kimiri	Control	19.30 lm	35.90 b	29.00 e-h	34.1b-d
	0.25% CaB	9.90 n	32.53 b-e	43.60 a	26.13hi
	0.2%KP	24.53 i-k	35.03 bc	26.43 hi	25.8 h-j
Khalal	Control	8.93 n	27.50 g-i	22.17 j-l	23.90ik
	0.25% CaB	30.93 d-g	35.67 b	33.50 b-d	31.7c-f
	0.2%KP	19.00 lm	32.27 b-e	34.53 b-d	25.5 h-j
**Fruit non-reducing sugars% in 2020 season**					
Fruit set	Control	19.60 m-o	32.33 c-e	29.07 e-h	32.93 cd
	0.25% CaB	18.70 no	19.60 m-o	17.73 o	28.33 g-i
	0.2%KP	21.07 l-n	26.33 h-j	21.83 l-n	20.10 m-o
Kimiri	Control	19.60 m-o	32.33 c-e	29.07 e-h	32.93 cd
	0.25% CaB	14.17 p	31.03 d-g	38.90 a	27.07 h-j
	0.2%KP	22.40 k-m	36.50 ab	26.37 h-j	25.50 i-l
Khalal	Control	9.63 q	27.20 hi	23.80 j-l	25.93 h-j
	0.25% CaB	28.80 f-i	34.60 bc	33.13 cd	31.30 c-g
	0.2%KP	19.20 m-o	31.77 c-f	33.30 b-d	26.93 h-j

The values following the same letter in each season do not differ significantly at a probability level of 0.05. CaB = calcium boron, KP = potassium phosphite.

K plays a necessary role in amino acids synthesis, sugar movements and assimilates besides carbohydrates accumulate^12^. Moreover, K regulates carbohydrate biosynthesis, cell water content, nitrogen uptake and translocation^13–15^. Foliar application of Ca and B was more effective than soil application^29–31^. B increase fruit sugar content through increasing the transport of carbohydrates through the association of OH groups in the sugars with the borate radical to facilitate their transfer into the plant, which leads to increasing the fruits TSS and sugars content as well as increase fruit dry matter^32,33^. These findings were align with Al-Saikhan^48^ who reported that thinning of one-third of the total number of strands, one-third of the length of the strands and thinning by both methods together increased reducing sugars and non-reducing sugars in the Ruzeiz date palm cultivar. Soliman and Hahash^46^ showed that 15 and 30% thinning of the strands resulted in higher total sugars, reducing and non-reducing sugars at bisr and tamr stages in the Succary date palms. Alebidi et al.^45^ found that 2% KNO_3_+1% algae extract resulted in increasing fruit reducing, non-reducing, and total sugars of the Barhee date palm.

Since thinning significantly reduces the bunches weight, but it is important for increasing export, keeping palm healthy and preventing alternate bearing. Properly fruit thinning while applying nutrients will yield the most favorable outcomes in this aspect.

Both fruit thinning and fruit spraying participate in improving fruit quality. Individual fruit thinning like removing 1/3 strands number, after fruit set successfully increased fruit weight and fruit length by 8.53% and 6%, respectively. Individual spraying of CaB and KP increased fruit weight by 6.80% and 17.11% and increased fruit length by 3.38 and 3.18% as mean of both seasons, respectively. Moreover, at Khalal stage, individual fruit thinning like removing 1/3 strands number succeeded in increasing fruit TSS by 0.93%, while at the same stage, individual CaB spraying increased it by 11.17% and individual KP spraying increased it by 11.64% as mean of both seasons, respectively. Moreover, at Khalal stage, individual fruit thinning like removing 1/3 strands number succeeded in increase fruit total sugars by 11.75%, while at the same stage, individual CaB increased it by 33.23% and individual KP spraying increased it by 13.69% as mean of both seasons, respectively.

At the Kimri stage, removing 1/3 strands number succeeded in increasing fruit weight, length, and size by 10.44%, 8.28%, and 15.68%, while incorporating spraying of CaB to removing 1/3 strands number led to increases of 43%, 26.78%, and 52.78%, while incorporating spraying of KP to removing 1/3 strands number resulted in increases of 42.67%, 26.31%, and 44.22%, compared to control, as mean of both seasons, respectively. At Khalal stage, combined removing 1/3 strands number and CaB increased fruit TSS and total sugars by 18.85% and 33.65%, as well as combined removing 1/3 strands number and KP increased it by 19.29% and 23.81%, respectively. Furthermore, at Kimri stage, removing 1/3 strands number and KP spraying increased fruit TSS and total sugars by 23.94% and 10.53% as well as combined removing 1/3 bunches number and CaB spraying increased it by 18.73% and 2.51% as mean of both seasons, compared to the un-thinned and un-sprayed control palms, respectively.

It can be concluded that spraying nutrients improved fruit chemical properties, thinning enhanced fruit physical properties. Furthermore, early spraying stage participates in improving fruit physical properties, thinning supplies more nutrients for the remaining fruits and combined methods give the best fruit properties.

##  Conclusions

Fruit thinning (removing 1/3 bunches number, removing 1/3 strands length or number) and spraying either calcium boron (CaB) or potassium phosphite (KP) at three fruit growth stages (after fruit set or Kimri or Khalal ) improved Medjool date fruit quality. At the Kimri stage, removing 1/3 of the strands number increased fruit weight, length, and size by 10.44%, 8.28%, and 15.68%. Supplemental CaB spraying improved these measurements to 43%, 26.78%, and 52.78%, while KP spraying achieved increases of 42.67%, 26.31%, and 44.22%. At the Khalal stage, combining removing 1/3 of the strands number with CaB spraying raised fruit TSS and total sugars by 18.85% and 33.65%, while KP spraying led to increases of 19.29% and 23.81%. Overall, nutrient spraying enhanced fruit properties, with early applications benefiting physical properties and later ones enhancing chemical properties. Thinning further improved these attributes.

## Data Availability

The data generated and/or analysed during the current study are available per request to the corresponding author.
